# Trends and estimated costs of hospitalizations potentially associated with respiratory syncytial virus among children in Brazil, 2014-2025

**DOI:** 10.1590/0037-8682-0120-2026

**Published:** 2026-09-18

**Authors:** Felipe Cotrim de Carvalho, Walquiria Aparecida Ferreira de Almeida, Daiana Araújo da Silva, Francisco José de Paula, Dalva Maria de Assis, Rafaela Gomes Andrade, Marcela Santos Corrêa da Costa, Marcelo Ferreira da Costa Gomes, Priscila Leal e Leite, Nelson J. Alvis-Zakzuk, Paula Couto, Jorge Jara, Paola Cristina Resende, Marilda M. Siqueira, Fernando do Couto Motta, Wildo Navegantes de Araújo, Mitermayer Galvão Reis

**Affiliations:** 1 Ministério da Saúde, Fundação Oswaldo Cruz, Instituto Gonçalo Moniz, Salvador, BA, Brasil.; 2 Ministério da Saúde, Secretaria de Vigilância em Saúde e Ambiente, Departamento de Doenças Transmissíveis, Coordenação-Geral de Vigilância da Covid-19, Influenza e Outros Vírus Respiratórios, Brasília, DF, Brasil.; 3 Fundação Oswaldo Cruz, Laboratório de Vírus Respiratórios, Enterovírus e Emergências Virais, Rio de Janeiro, RJ, Brasil.; 4 Universidade de Brasília, Medicina Tropical, Brasília, DF, Brasil.; 5 World Health Organization, Department of Public Health Emergencies, Pan American Health Organization, Washington, DC, USA.; 6 Universidad de la Costa, Department of Health Sciences, Barranquilla, Colombia.; 7 World Health Organization, Pan American Health Organization, Comprehensive Immunization Program, Washington, DC, USA.; 8 Universidade Federal da Bahia, Faculdade de Medicina da Bahia, Salvador, BA, Brasil.; 9 Yale University, Department of Epidemiology of Microbial Diseases, School of Public Health, New Haven, CT, USA.

**Keywords:** Respiratory syncytial virus, bronchiolitis, hospitalization, health care costs, seasonality, Brazil

## Abstract

**Background:**

Respiratory syncytial virus, a major cause of respiratory infections and hospitalizations in young children, especially bronchiolitis, is an important burden to the health system in Brazil. This study evaluated the trends, seasonality, and economic impact of hospitalizations associated with respiratory syncytial virus in Brazilian children.

**Methods:**

This study analyzed hospitalizations for acute bronchitis and bronchiolitis (International Code of Diseases-10 J20-J21) in Brazilian children <5 years of age from January 2014 and March 2025 using data from the Brazil’s Unified Health System. Annual trends were assessed with linear regression, while time series decomposition evaluated seasonality. Direct medical costs were adjusted for inflation and converted to international dollars (Int$). Pearson’s correlation coefficient was employed to analyze associations between length of stay and hospitalization cost.

**Results:**

A total of 727,808 hospitalizations were recorded, with 86.8% occurring in children under 5 years old. Hospitalizations increased by 307.8%, with marked declines in 2020 during the coronavirus disease-2019 pandemic and consistent seasonal peaks between March**-**June. Direct medical costs totaled Int$ 166.8 million, with 55.5% of expenditures after 2020. The mean cost per hospitalization increased by Int$ 21.48 between 2014-2024 (Int$ 118.16-139.64). Length of stay correlated moderately with cost (*r* = 0.55), particularly strongly in southeastern and southern Brazil.

**Conclusions:**

Our findings revealed distinct seasonal and regional patterns in hospitalizations and a post-pandemic increase in costs. The association between prolonged hospitalization and higher expenditures underscores the need for targeted, region-specific preventive strategies and timely clinical interventions, to reduce the growing economic burden on the system.

## INTRODUCTION

Respiratory syncytial virus (RSV) is a major cause of respiratory infections in children, often associated with pneumonia and bronchiolitis^1^. Bronchiolitis caused by RSV constitutes the leading cause of hospital admissions among children aged<2 years^2^. Globally, RSV accounts for significant morbidity and mortality related to respiratory infections, mainly affecting children^1^. Similar to other respiratory viruses, it exhibits a seasonality characterized by variability in incidence within and among regions of the same country^3^.

Hospitalizations due to RSV impose a considerable economic burden on health systems, families, and society^4^, especially in middle-income countries^5^. Reportedly, hospitalization rates have increased in recent decades, highlighting escalating resource utilization, rising costs, and a growing public health impact^6^. The significant clinical and economic burden of bronchiolitis hospitalizations has driven the development of active and passive immunizations, especially in high-risk infants^7^.

Despite these preventive interventions, national-level data on the epidemiological trends and medical costs of RSV-associated hospitalizations in Brazil remain scarce. Such data are essential to support cost-effectiveness analyses and guide the prioritization of immunization strategies within the Brazilian Unified Health System (BUHS).

Given this context, we evaluated the economic impact of hospitalizations from RSV within the BUHS and their relationship with seasonality. We examined temporal variations in the incidence and direct medical costs of RSV-associated hospitalizations across different regions in Brazil. 

## METHODS

### Study design

This study analyzed the trend in hospitalizations caused by RSV from 2014 to 2025^8^, estimating the direct medical costs based on monthly data grouped by Brazil’s geographical regions.

The Brazilian macro-region (North, Northeast, Midwest, Southeast, and South) was used as the stratification variable, with data aggregated at the regional level by month of hospital admission and patient's region of residence.

### Data curation

Data were extracted on May 11 from the BUHS’s Hospital Information System (SIH) via the DATASUS TabNet tool using the filters “hospital morbidity” and “place of residence”, covering the timespan of January 2014 to March 2025 by region and federative unit^9^. The analysis included hospitalizations of children under 5 years of age (0-4 years), considering all admission types (elective or urgent) and care regime (public or private contracted to the BUHS). Hospitalizations financed exclusively by private health insurance or out-of-pocket payment are not recorded in SIH and are therefore not captured in this analysis. International Code of Diseases (ICD)-10 codes J20-J21 (bronchitis and bronchiolitis) were selected^10^. Case definition was based solely on ICD-10 administrative coding, without laboratory confirmation; therefore, RSV cannot be directly attributed to individual hospitalizations, and the term 'potentially associated with RSV' is used throughout to reflect this limitation. Data were exported as .csv files and processed using R Foundation for Statistical Computing, Viena, Áustria, for organization, cleaning, and aggregation to support the statistical and temporal analyses^9^ ^,^ ^10^. 

Data extracted for the period January-March 2025 were considered preliminary, as records from the last 6 months in SIH/DATASUS are subject to consolidation updates; these data though included in descriptive and cost analyses, should be cautiously interpreted.

### Time series analysis: hospitalization’s annual growth rate

The annual growth rate was estimated using a simple linear regression model (Equation 1):

Y=β0+ β1X+ε (1)

where Y is the number of monthly hospitalizations, X is time (in months), is the intercept, β1 is the mean monthly increase or decrease rate (annualized estimates in the Results were calculated as 12×β1), and ε is the error term.

The analyses were conducted using the R software^11^, using the core packages (I) ggplot2^12^ for graphical display, (II) dplyr^13^ for data manipulation, (III) readxl^14^ for reading Excel files, (IV) patchwork^15^ for combining graphics, combination, and (V) lubridate^16^ for date manipulation. The trend line from linear regression was automatically adjusted with the geom_smooth (method = “lm”) function in ggplot2^12^, which estimated the coefficients (β0 and β1) and illustrated the fitted line.

### Time series decomposition

Following linear analysis, time series decomposition was performed^17^ ^,^ ^18^. Data were aggregated by month for each region and converted into time series objects (ts) in R software via the stats package^11^. Each series was assigned a monthly frequency (12 per year), spanning January 2014 to December 2024 (complete annual cycles; the three months of partial 2025 data were excluded from the decomposition to avoid distortion of the seasonal component, while descriptive and cost analyses include data through March 2025). The decomposition differentiated the trend, seasonal, and residual components. Seasonal factors were examined to characterize and validate temporal patterns. Decomposition was performed with the decompose function (stats package)^11^ for each regional and national time series. Graphs were generated using the ggplot2^14^ autoplot function and organized to emphasize regional disparities.

Autocorrelation analysis^19^ ^,^ ^20^ was employed to detect temporal dependencies and seasonality in Brazil and its regions, using the acf function from the stats package^11^. It measured correlations at different lags and graphs were generated through the ggAcf function (forecast package)^21^, to identify short-term dependencies and seasonal or cyclical patterns across time scales.

### Direct medical cost estimation

Direct medical costs of hospitalizations caused by RSV were estimated for 2014-2025. Mean costs per hospitalization were calculated annually for Brazil and each of its five regions. Boxplots were generated using ggplot2 (geom_boxplot)^12^ to illustrate the annual dispersion of mean hospitalization costs. Descriptive statistics, including standard deviation and variance of mean values, were calculated for each year and region using dplyr functions^13^.

The correlation between mean length of stay and mean direct medical cost per hospitalization was estimated using the cor function (stats package)^11^. A simple linear regression model (lm function) was applied to assess the association between average length of stay (independent variable) and adjusted average cost per hospitalization (dependent variable), corrected for inflation and converted to international dollars (Int$) using the purchasing power parity (PPP) conversion factor of 2.524 BRL/Int$, as recommended by the Organisation for Economic Co-operation and Development and the World Bank^22^ ^,^ ^23^. 

Pearson’s correlation coefficient was employed to measure the strength and direction of the linear relationship between mean length of stay and mean direct medical cost per hospital stay^24^ ^,^ ^25^. Scatter plots were generated for each region, with trend lines based on the fitted regression model using geom_point function to plot the scatter points and geom_smooth (method = “lm”) from ggplot2^14^ to add the regression line. Correlation strength was interpreted according to conventional thresholds.

### Inflation adjustment and currency conversion

To ensure temporal comparability, hospitalization costs were adjusted for inflation using Brazil’s extended national consumer price index provided by the Brazilian Institute of Geography and Statistics (SIDRA table 1737, national level)^26^. Monthly values were aggregated by calendar year, and the national average for 2024 was adopted as the reference base year.

For international comparability, inflation-adjusted values were converted to Int$ using Brazil’s 2024 PPP conversion factor of 2.524 BRL/Int$, as published by the World Bank^23^. A fixed PPP factor was utilized to present the historical series in standardized purchasing power, reflecting the actual economic burden of hospitalization costs in Brazil. All PPP conversions were applied exclusively to inflation-adjusted BRL values, following international best practices^22^. 

### Ethical considerations

This study was approved by the research ethics committee of the Gonçalo Moniz Research Center (approval no. 6.929.375 and IORG0002090/OMB no. 0990-0279) and complied with the Brazilian National Health Council Resolution CNS no. 466/2012. The databases included anonymized information from the Hospital Information System and the Mortality Information System, accessed via TABWIN and TABNET platforms (DATASUS) under e-SIC protocols no. 25072.028465/2024-98 and no. 25072.017378/2024-13.

## RESULTS

**FIGURE 1:**
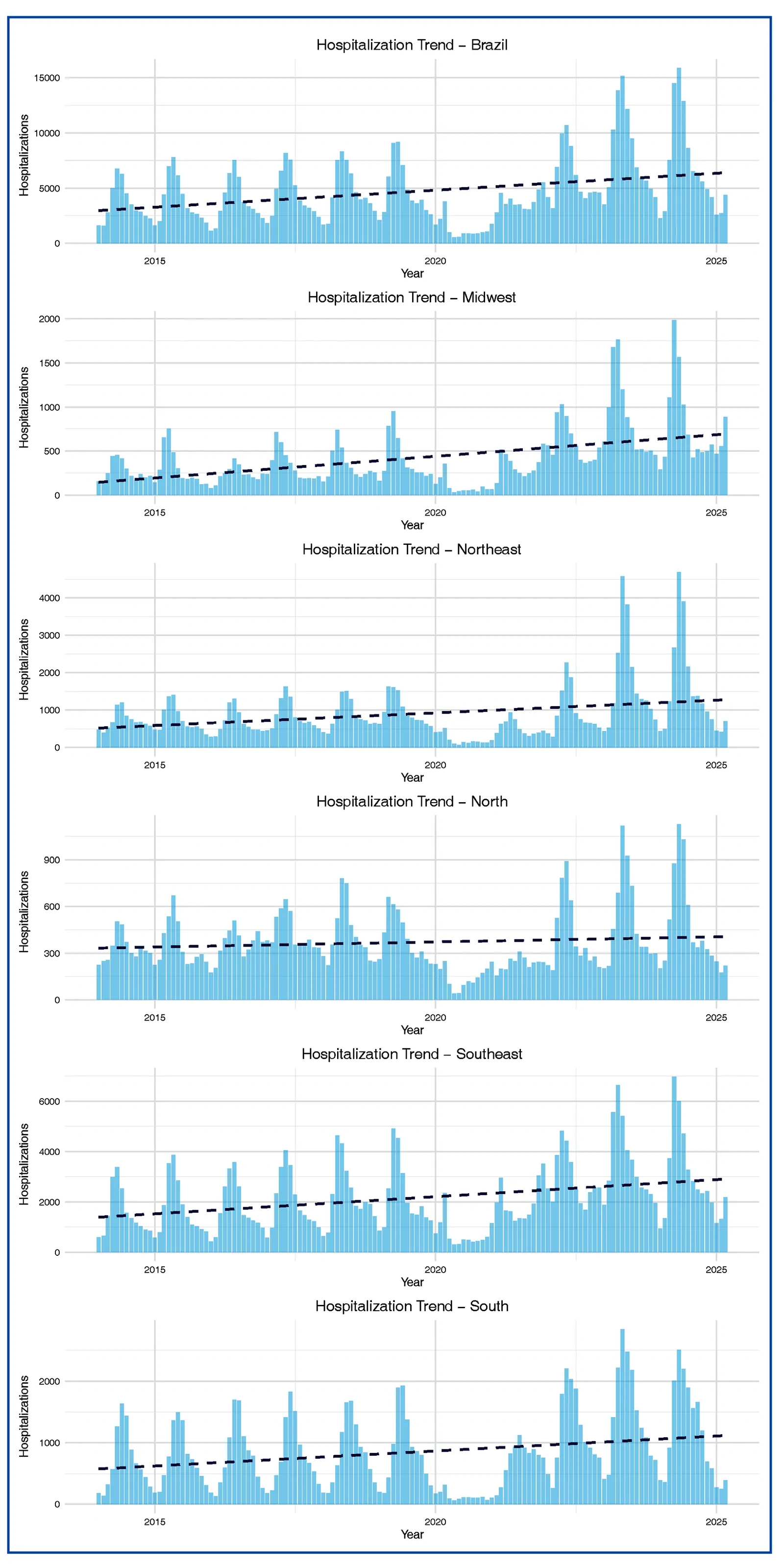
Trend in hospitalizations for bronchitis and acute bronchiolitis by month and year of care (2014-2025). **Source:** Brazilian Ministry of Health^12^. Data for the last 6 months, subject to change.

Between January 2014 and March 2025, Brazil reported 727,808 hospitalizations for acute bronchitis and acute bronchiolitis; of these, 86.8% (n = 631,724/727,808) occurred in children < 5 years of age. From 2014 to 2024, total hospitalizations increased by 307.8% (Figure 1). The southeastern region showed the highest increase (135.9%), followed by the northeastern (67.9%), midwestern (48.9%), and southern (48.5%) regions; northern Brazil had the lowest increase (6.6%). There was a marked national decline in 2020, corresponding to the first year of the coronavirus disease-2019 (COVID-19) pandemic (Figure 1).

Overall, hospitalizations rose significantly (128,6%) from 3548 in July 2014 to 8110 in June 2023. Residuals reflected variations such as the abrupt decline at the start of the COVID-19 pandemic (-3293 in April 2020) and isolated spikes (1742 in June 2023), a trend present in all regions. Seasonality remained stable, with a consistent annual cycle across regions (Supplementary Figure 1). 

In the midwest region, hospitalizations increased by 207% (from 265 in July 2014 to 814 in September 2024), with a decrease of 489 cases in April 2020, potentially attributed to the pandemic. In the northeast, the trend rose 143% (from 715 to 1740), also with notable residuals in 2020. The north experienced a more modest growth (54%; from 331 to 511), with reductions in May 2020 (- 353) and increases in May 2023 (+ 383). In the southeast, hospitalizations increased 108% (from 1538 in July 2014 to 3205 in September 2024), with residuals reducing in April 2020 (- 2113) and growing in March 2023 (+ 1266). In the south, cases grew from 700 in January 2014 to 1290 in September 2024, with residuals declining in June 2020 (- 840) during the pandemic (Supplementary Figure 1). Autocorrelation analysis indicated a seasonality at lag 12 (ACF = 0.60) in Brazil. The southeast (0.60) and south (0.64) had the strongest annual seasonality, followed by the midwest (0.57) and northeast (0.59). The north showed a weaker pattern (0.52) (Supplementary Figure 2).

**FIGURE 2:**
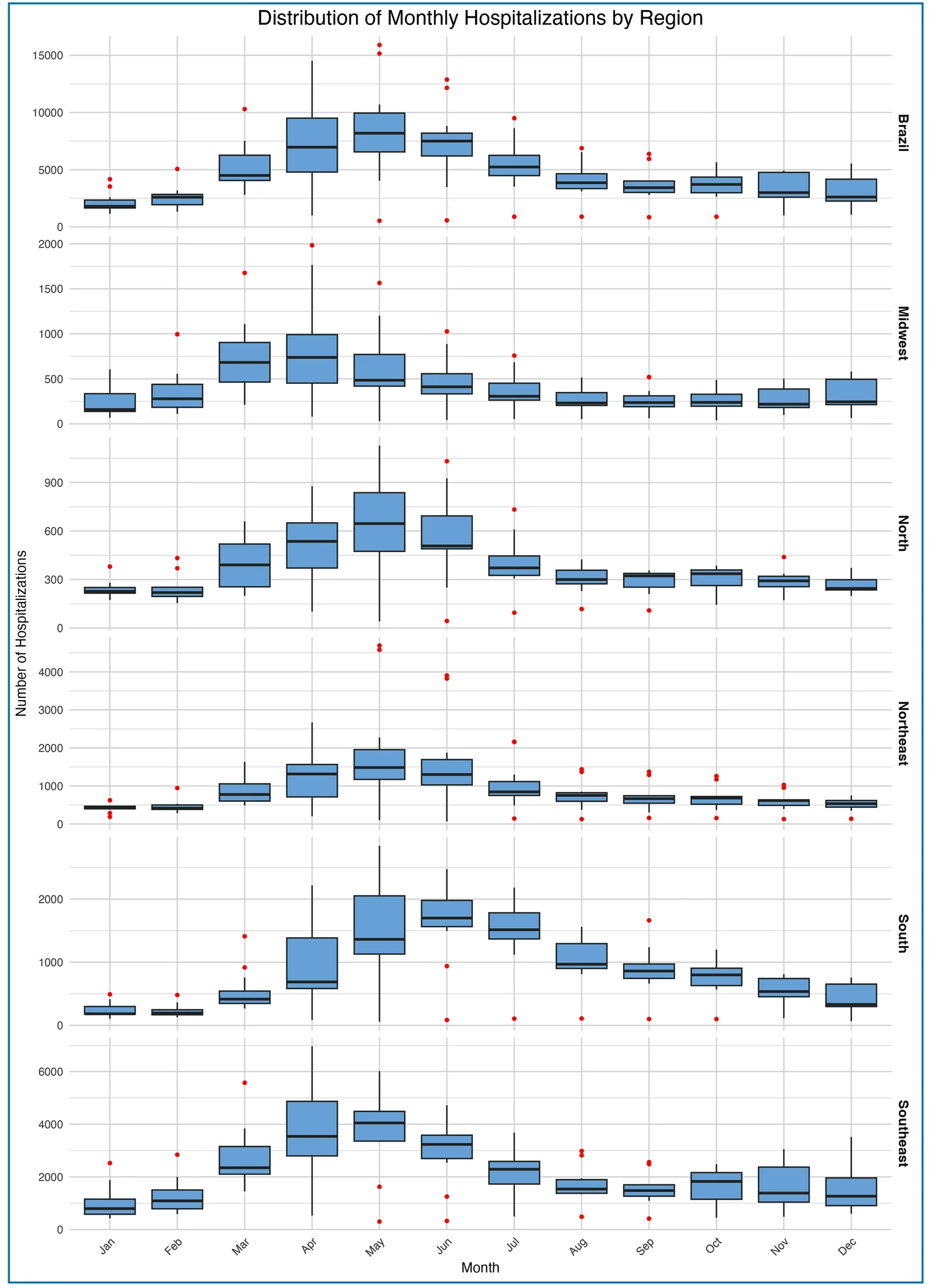
Hospitalizations for bronchitis and acute bronchiolitis potentially associated with respiratory syncytial virus cases by month and year (2014-2025). **Source:** Brazilian Ministry of Health^12^. Data for the last 6 months, subject to change.

Regional boxplots exhibited a consistent seasonality, with hospitalization peaks between March and June. April and May had the highest median values in most regions. Nationally, the peak occurred between March and July, suggesting a consolidated epidemic pattern. In the northeast, north, and midwest, hospitalizations increased from March to June. In the south and southeast, seasonality persisted longer, with elevated rates until mid-August. From August to December, all regions showed lower medians and interquartile ranges, expressing reduced hospitalization rates (Figure 2).

Between 2014-2025, hospitalizations for acute bronchitis and bronchiolitis in children aged <5 years resulted in Int$ 166.8 million. From 2020 to 2024, Int$ 92.5 million was spent (approximately 55.48% of the total), a 29.1% increase compared to that in the pre-pandemic period (2014-2019), when expenditures totaled Int$ 71.7 million, underscoring the substantial economic impact of the post-COVID-19 period on pediatric respiratory hospitalizations (Table 1).

**TABLE 1:** Hospitalizations and direct costs associated with acute bronchitis and acute bronchiolitis in children < 5 years old by year of occurrence (2014 to March 2025).

Year	Hospitalizations^*^	Nominal cost (BRL)^**^	IPCA adjusted cost (BRL)^†^	Cost adjusted for inflation using PPP (Int$)^‡^	Adjusted cost average (Int$)^§^
2014	42593	15.424.520.43	27.125.787.47	10.747.142.42	252.32
2015	46145	17.369.707.95	28.016.738.92	11.100.134.28	240.55
2016	44997	17.185.039.31	25.491.165.27	10.099.510.80	224.45
2017	52560	21.340.848.80	30.600.997.12	12.124.008.37	230.67
2018	56487	24.853.209.05	34.377.543.25	13.620.262.78	241.12
2019	58145	26.506.302.64	35.344.730.79	14.003.459.11	240.84
2020	15358	7.607.614.01	9.828.670.54	3.894.085.00	253.55
2021	44011	26.661.821.30	31.805.410.78	12.601.192.86	286.32
2022	72475	46.459.731.93	50.716.209.54	20.093.585.40	277.25
2023	97119	68.900.536.70	71.909.742.88	28.490.389.41	293.36
2024	92144	69.353.076.96	69.353.076.96	27.477.447.29	298.2
2025	9690	6.732.678,25	6.492.094,91	2.572.145,37	265.44

**PPP:** Purchasing power parity. ^*^Number of hospitalizations during the year; ^**^total hospitalization costs by year in BRL; ^†^annual cost (BRL) spent on hospitalizations adjusted for inflation; ^‡^annual cost of hospitalizations, adjusted for inflation and converted into international dollars (Int$) based on PPP; ^§^mean cost of hospitalization in a given year, calculated by the ratio of total hospitalization expenditures to the total number of hospitalizations in the same year, with the values adjusted for inflation and expressed in international dollars (Int$) based on PPP. **Source:** The Brazilian Ministry of Health^12^. Data for the last 6 months, subject to change.

**FIGURE 3:**
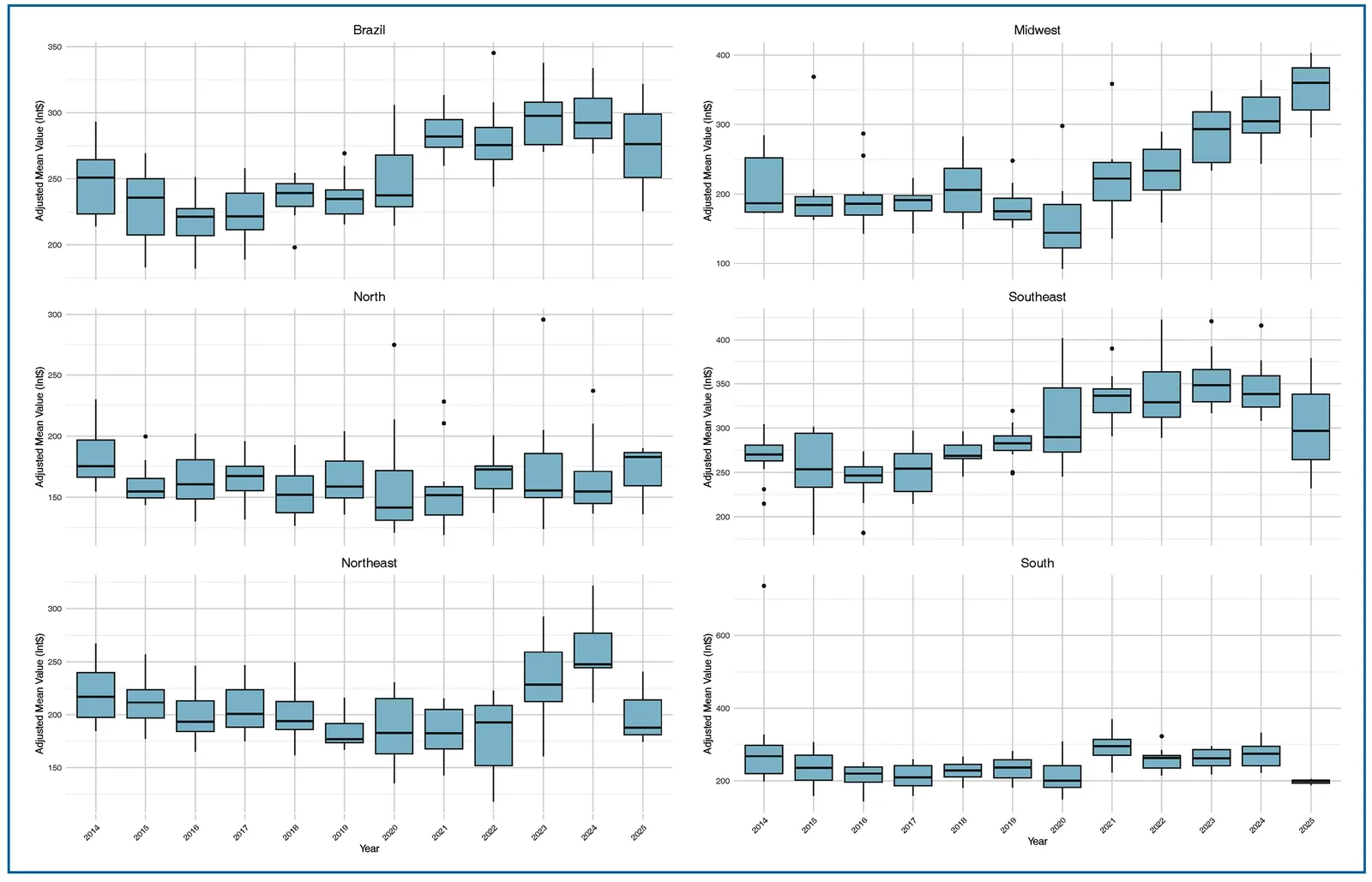
Average cost dispersion of hospitalizations for acute bronchitis and acute bronchiolitis in children < 5 years old, adjusted for inflation and converted to international dollars (Int$) per year (2014 to March 2025). **Source:** Brazilian Ministry of Health^11^. Data for the last 6 months, subject to change.

From 2014 to 2024, the national average direct medical cost per hospitalization increased by 18.2% from Int$ 252.32 to Int$ 298.20, with an average of Int$ 258.06, showing a consistent upward trend in hospital expenditures on acute respiratory conditions in early childhood (Table 1; Figure 3).

In the midwestern and northeastern regions, the mean cost increased from Int$ 210.51 to 308.44 (46.5%) and from Int$ 219.66 to 261.28 (18.9%), respectively, with average cost being Int$ 227.77 and 205.10, respectively, thus exhibiting moderate and sustained growth in these regions. Contrary to the national trend, the north presented a decline in costs, decreasing from Int$ 181.60 to 164.45 (- 9.4%). The mean cost for the period (Int$ 164.75) was the lowest among all regions, indicating unique regional expenditure trends for pediatric respiratory hospitalizations. The southeast exhibited the highest hospitalization costs, rising from Int$ 267.62 to 343.35 (+ 28.3%). The mean cost across the 2 years was Int$ 295.92. Hospitalization costs reduced in the south from Int$ 299.29 to 272.16 (-9.1%). Still, the mean cost over the period was Int$ 242.81, positioning the region among those with higher expenditures nationally, albeit below the southeast. Preliminary data for 2025 are consistent with previous trends. Nationally, the average adjusted cost per hospitalization was Int$ 274.55, exceeding the 2024 value. The midwest recorded the highest value (Int$ 348.21), while the north maintained the lowest (Int$ 169.62), highlighting persistent regional disparities (Figure 3).

In Brazil, length of stay correlated moderately and significantly with direct medical costs (*r* = 0.55; *p* < 0.001; R^2^ = 0.305), explaining approximately one-third of cost variation. The midwest exhibited the strongest correlation (*r* = 0.61; *p* < 0.001; R^2^ = 0.367), with a regression coefficient of Int$ 80.57 per additional day. In the southeast, moderate-to-high correlation was observed (*r* = 0.59), with the largest daily cost increase (Int$ 90.88). The south also demonstrated a strong association (*r* = 0.60; *p* < 0.001; R^2^ = 35.6%), with a daily average increase of Int$ 72.45. In the northeast, correlation was lower (*r* = 0.50; *p* < 0.001), with a daily increase of Int$ 51.70, similar to the national trend but with less impact. The north had the weakest correlation (*r* = 0.47; *p* < 0.001; R^2^ = 22.5%), although significant, with an additional Int$ 33.63 per additional day (Supplementary Figure 3; Table 2).

**TABLE 2:** Correlation between length of stay and hospitalization costs by region (2014 to March 2025).

Region	**Correlation (*r*)**	Regression coefficient (Int$/day)	R^2^	*p*
Brazil	0.55	68.28	30.5	< 0.001
Central-west	0.61	80.57	36.7	< 0.001
Northeast	0.50	51.7	25.2	< 0.001
North	0.47	33.63	22.5	< 0.001
Southeast	0.59	90.88	34.8	< 0.001
South	0.60	72.45	35.6	< 0.001

*p* < 0.001 based on scientific notation values; R^2^ values were rounded to one decimal place for clarity. **Source:** Brazilian Ministry of Health^12^. Data for the last 6 months, subject to change.

## DISCUSSION

This study evaluated the growing health and economic burden of hospital admissions for RSV-associated bronchiolitis and acute bronchitis in Brazil from January 2014 to March 2025. Nationwide, hospitalizations increased substantially, particularly among children under 5 years, a highly vulnerable group. These findings align with previous research comparing pre- and post-COVID-19 pandemic periods^27^.

Notably, a reduction in bronchiolitis hospitalizations occurred at the onset of the COVID-19 pandemic in 2020, consistent with declines reported for influenza and other respiratory viruses^27^. Reportedly, social distancing measures and widespread mask use contributed to the reduced transmission of respiratory viruses, including RSV^28^; closure of schools and daycare centers also contributed^29^. As documented in Brazil and globally, the pandemic also disrupted healthcare continuity: reducing outpatient visits, delaying diagnoses, and decreasing hospital admissions for non-COVID conditions. The subsequent overload of health services during the post-pandemic rebound period may have further contributed to more severe presentations at hospital admission. Nevertheless, the subsequent rise in hospitalizations during 2023 and 2024 highlights the effects of resuming regular activities and the susceptibility of children with no prior exposure to the virus. This phenomenon, described in the literature as "immunity debt," refers to the accumulation of immunologically naïve individuals during the pandemic period, resulting in larger and potentially more severe RSV epidemic waves upon viral reintroduction. The increased mean daily hospitalization costs observed in the southeast and midwest post-2020 may partly reflect a higher proportion of clinically severe cases in this unexposed cohort, consistent with the immunity debt hypothesis^29^. This trend also reflects enhanced sensitivity in surveillance systems and improved recording of respiratory infections during this period.

In time series decomposition, residual variance increased in all regions after the COVID-19 pandemic, revealing greater sensitivity in hospitalization data. This may result from improvements in case detection, reporting, monitoring, diagnostics, digital health infrastructure, and service protocols, which improved data granularity and responsiveness and contributed to the higher residuals in the post-COVID era²⁷.

Monthly boxplots demonstrated consistent seasonality, with hospitalizations peaking from March to June, most pronounced in the southeast and south. In the south, the seasonality extended until August, indicating a longer epidemic window that demands additional preventive efforts. Freitas and Donalisio^30^ have described distinct regional peaks of RSV activity across Brazilian regions, with longer epidemic in the south and southeast, extending into August. Similarly, Wollmeister and collaborators^31^ reported a shift in peak activity in April in southeastern Brazil, demonstrating a broader temporal window of viral circulation and heightened hospital demand. The increase in cases after 2020 suggests a post-pandemic shift in hospitalization profiles, likely driven by more complex cases and reduced immunity among children.

The regional seasonality patterns: prolonged epidemic windows in the south and southeast and earlier peaks in the north, are consistent with the established role of climatic factors in driving RSV transmission. Temperature, humidity, and precipitation are key determinants of RSV seasonality, and climate change projections suggest that rising temperatures may alter the amplitude, timing, and geographic distribution of outbreaks, potentially extending epidemic windows in tropical regions such as northern Brazil. These findings reinforce the need for adaptive surveillance systems and immunization calendars that incorporate climatic projections into RSV prevention planning^32^.

Greater intra-annual variability during peak months, in addition to higher monthly medians, infer that while seasonality is consistent, outbreak magnitude varies across years. Significant outliers in the southeast and south underscore epidemic years that can strain hospital services. These findings emphasize the need for continuous surveillance and anticipatory planning, with strengthened resource allocation during high-risk periods to improve responses to respiratory virus seasonality.

Average length of stay correlated positively with hospitalization costs. This association was evident nationally, with the strongest effects in the midwest and southeast; the north exhibited the weakest correlation. These observations align with previous studies demonstrating that prolonged intensive care unit stays for infants with RSV substantially increase hospitalization costs^33^ ^,^ ^34^.

Although positive association between length of stay and hospitalization cost is expected, quantifying this relationship is essential to reinforce its relevance in various regional contexts. The correlations express differences in explanatory power, reflecting disparities in care models, clinical practices, and system efficiency. These findings support implementing strategies to reduce unnecessary hospital days, improve outcomes, and optimize costs.

The substantial increase in hospitalizations observed nationally was accompanied by a parallel rise in direct medical expenditures. During the study period, over Int$ 166.82 million were spent on hospitalizations for bronchiolitis and acute bronchitis among children, with 55.5% of this total concentrated in the post-pandemic period (2020-2024), reflecting both the surge in case volume and the higher mean costs per hospitalization observed after 2020. These estimates exclude indirect costs or out-of-pocket expenditures, which would increase the total economic burden. Although this analysis did not identify specific causes of increased hospitalization costs, the results reinforces the economic burden associated with acute bronchiolitis and highlight the need for preventive measures, such as vaccination of pregnant women and passive immunization with monoclonal antibodies. These interventions could substantially reduce morbidity and mortality due to RSV^35^, consequently decreasing hospitalization costs.

Reportedly, laboratory-confirmed RSV hospitalizations declined by 50% among infants born to vaccinated mothers^36^. Complementary real-world data from the Centers for Disease Control and Prevention and the Food and Drug Administration indicated a 68% to 82% reduction in RSV-related hospitalizations during the first 3 months of life^36^ ^-^ ^38^. Collectively, these findings underscore the potential of maternal vaccination to provide early protection during the most vulnerable period of childhood, with potential long-term benefits to reduce both clinical complications and health care costs associated with RSV.

A pragmatic trial, which evaluated the safety and efficacy of the novel single-dose monoclonal antibody, demonstrated that nirsevimab reduced hospitalizations due to lower respiratory tract infection by 83.2%, a result corroborated by trials conducted in previously uninfected infants^39^ ^-^ ^41^. 

Importantly, nirsevimab was officially incorporated into the Unified Health System in 2025 in Brazil, with rollout beginning in February 2026 for preterm infants and children with comorbidities. Additionally, the maternal RSV vaccine received a favorable recommendation from CONITEC. These policy developments underscore the timeliness of the present study: the cost estimates reported here provide a pre-implementation baseline for future cost-effectiveness evaluations of these preventive technologies within the BUHS.

The northern region presented a distinct pattern: declining mean hospitalization costs (-9.4%) in contrast to the national upward trend. This suggests structural differences in BUHS reimbursement rates historically applied to Northern states, potential underreporting or less complete cost recording in SIH/DATASUS data from this region, and differences in case complexity or availability of specialized pediatric care infrastructure, thereby limiting intensive care access and reducing recorded cost intensity. Further investigation using disaggregated facility-level data is warranted to determine whether this trend reflects genuine gains in efficiency or limitations in data completeness.

In southern Brazil, hospitalization costs for bronchitis and bronchiolitis lowered between 2014-2024, contrasting with the national trend of rising costs. This decrease may reflect regional efficiencies in clinical management, including shorter stays and optimized care pathways. Historical strengths in primary health care and health indicators in the region may have contributed to less severe cases requiring less intensive hospital care. Nevertheless, the south maintained one of the highest mean costs over the period (Int$ 242.81), alluding to a persistent financial burden. These results warrant further investigation to determine whether reduced costs results from improved efficiency, data recording variations, or changes in reimbursement practices.

Our findings reinforce implementation of public policy to prevent RSV-associated bronchiolitis, particularly in the north and northeast. Effective resource allocation, combined with prevention measures targeting high-risk groups, is essential to mitigate the clinical and economic impacts of RSV. Despite the comprehensive analysis of hospitalization trends and direct medical costs associated with RSV-associated bronchiolitis in Brazil, specific knowledge gaps remain. Future studies should explore regional disparities, the effectiveness of different immunization strategies, and the relationships between climatic factors and seasonality. Moreover, it is also necessary to investigate whether variations in public health investment have influenced hospitalization rates, especially in regions with fewer resources. These analyses are essential for informing the development of effective public health policies.

This study’s limitations stem from the use of secondary administrative data from the Hospital Information System, a database primarily designed for reimbursement purposes, which may introduce coding biases, underreporting, and regional inconsistencies. The dataset lacks clinical variables (e.g., comorbidities, severity, readmissions), and diagnoses were based on ICD-10 codes (J20-J21) rather than on laboratory confirmation. ICD-10 codes J20-J21 capture the clinical syndromes of acute bronchitis and bronchiolitis, which may be caused by multiple respiratory pathogens, including rhinovirus, human metapneumovirus, and parainfluenza virus, in addition to RSV; the costs presented therefore reflect the economic burden of the clinical syndrome rather than exclusively of RSV-specific hospitalizations. These limitations introduce potential coding bias (misclassification of bronchiolitis etiology in the absence of virological data), underreporting bias (SIH captures only BUHS -financed care, excluding private health insurance and out-of-pocket hospitalizations), and selection bias (cases managed exclusively in the private sector were not represented). Cost estimations employed a single 2024 PPP conversion factor and were restricted to direct medical expenses, excluding indirect costs. As an ecological analysis, the results describe population-level trends and do not permit individual-level inference. Data for 2025 are preliminary; potential changes in hospital financing or care protocols could have influenced the cost patterns. 

In conclusion, this study identified significant epidemiological and economic trends related to RSV-associated hospitalizations in Brazil between 2014 and 2025. A consistent seasonal pattern emerged nationally, with peak hospitalizations between March and June and an extended epidemic window through August in the south and southeast. The post-pandemic period demonstrated a substantial rebound in both case volume and direct medical costs, with over 55% of the total Int$ 166.8 million expenditure concentrated after 2020. The strong association between length of stay and hospitalization costs across all regions underscores the economic value of early clinical intervention and case severity reduction. These findings provide a national pre-implementation baseline for evaluating the cost-effectiveness of recently incorporated preventive technologies in Brazil, including nirsevimab for high-risk infants and maternal RSV vaccination, both of which received favorable recommendations within the BUHS. Region-specific immunization calendars, adjusted to the distinct seasonal peaks identified, and sustained investment in primary care infrastructure, particularly in the Northern and Northeastern regions, are essential to reduce the growing burden of RSV on the BUHS. Future research should focus on the linkage between SIH and Influenza Epidemiological Surveillance Information System (SIVEP-Gripe) laboratory data to enable RSV-specific attribution, and on the long-term cost-effectiveness of the newly incorporated immunization strategies across Brazil.

**Disclaimer:** The content of this article is solely the responsibility of the authors and does not necessarily represent the official views or positions of the Oswaldo Cruz Foundation Fiocruz, the Brazilian Ministry of Health, the Pan American Health Organization/World Health Organization, or the University of Brasilia. The findings and conclusions expressed here are those of the authors and do not reflect the official policies or positions of the institutions to which they are affiliated.

## Data Availability

All aggregated data analyzed in this study are publicly available through the Brazilian Unified Health System’s Hospital Information System and can be accessed via the TABNET/DATASUS platform. Processed and summarized data supporting the findings are included in the article and its supplementary materials. Requests for access to more detailed or disaggregated data, including statistical code and data dictionary, may be made to the corresponding author, subject to ethical and legal considerations.
