# Supplementary material for: Trends and estimated costs of hospitalizations potentially associated with respiratory syncytial virus among children in Brazil, 2014-2025
Source: Rev Soc Bras Med Trop. 2026 Sep 18;59:e0120-2026. doi: 10.1590/0037-8682-0120-2026 (PMC13592856; doi:10.1590/0037-8682-0120-2026)
Supplement: Supplementary file 1 [file 1678-9849-rsbmt-59-e0120-2026-md1.pdf]

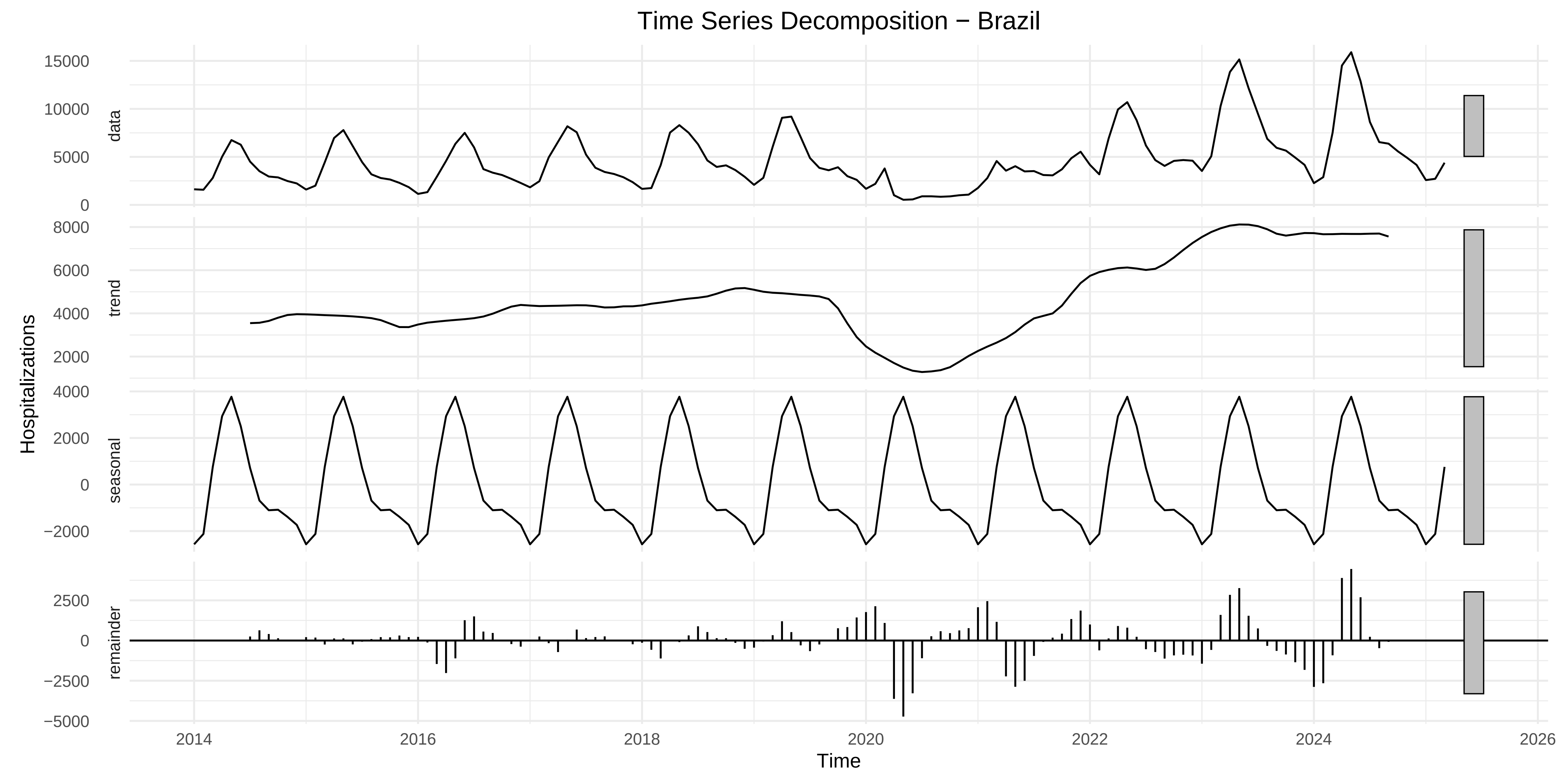
Time Series Decomposition − Brazil
Hospitalizations
data
trend
seasonal
remainder
15000
10000
5000
0
8000
6000
4000
2000
4000
2000
0
−2000
2500
0
−2500
−5000
2014
2016
2018
2020
2022
2024
2026
Time

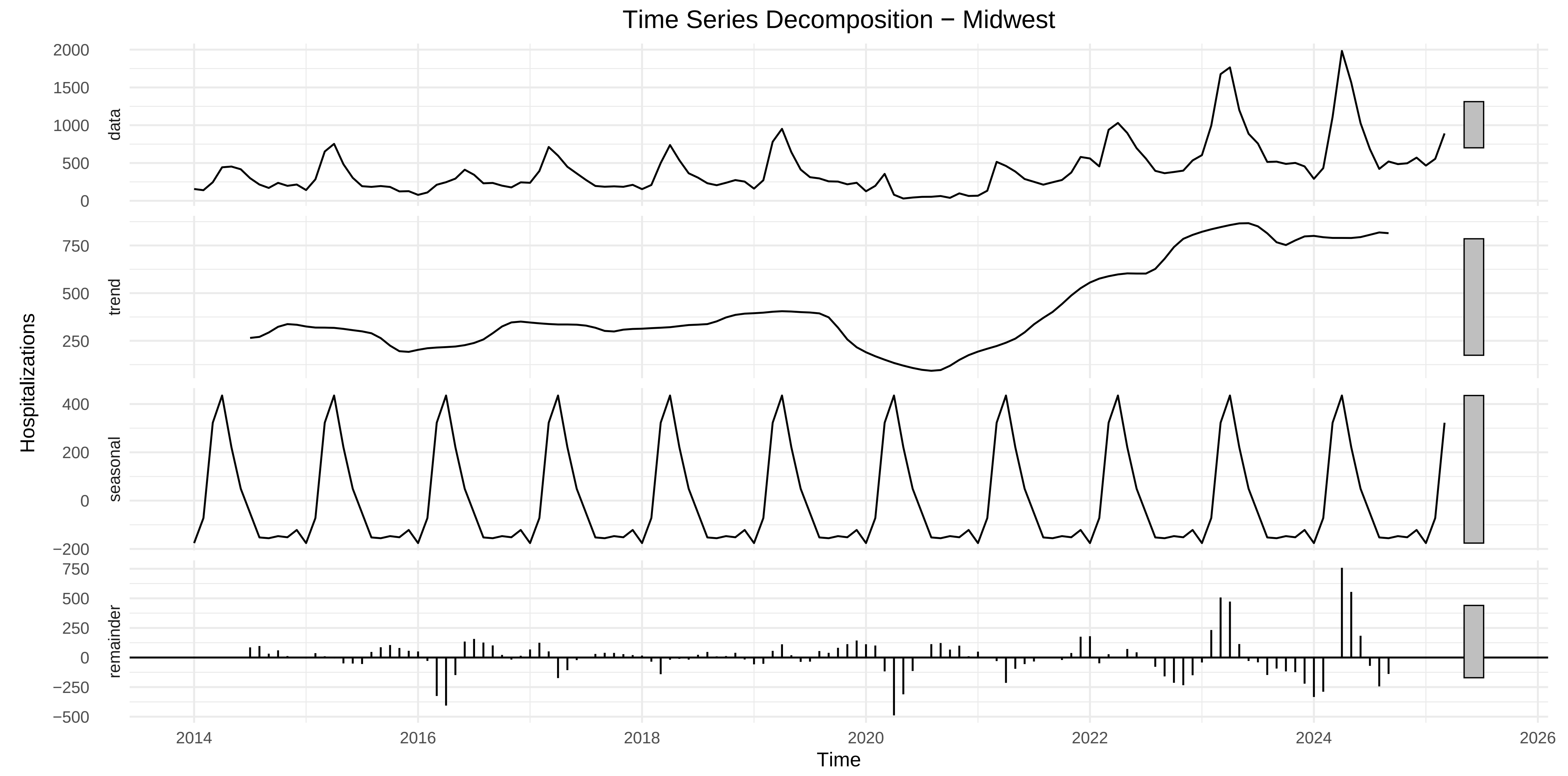
Time Series Decomposition − Midwest
Hospitalizations
data
trend
seasonal
remainder
2000
1500
1000
500
0
750
500
250
400
200
0
−200
750
500
250
0
−250
−500
2014
2016
2018
2020
2022
2024
2026
Time

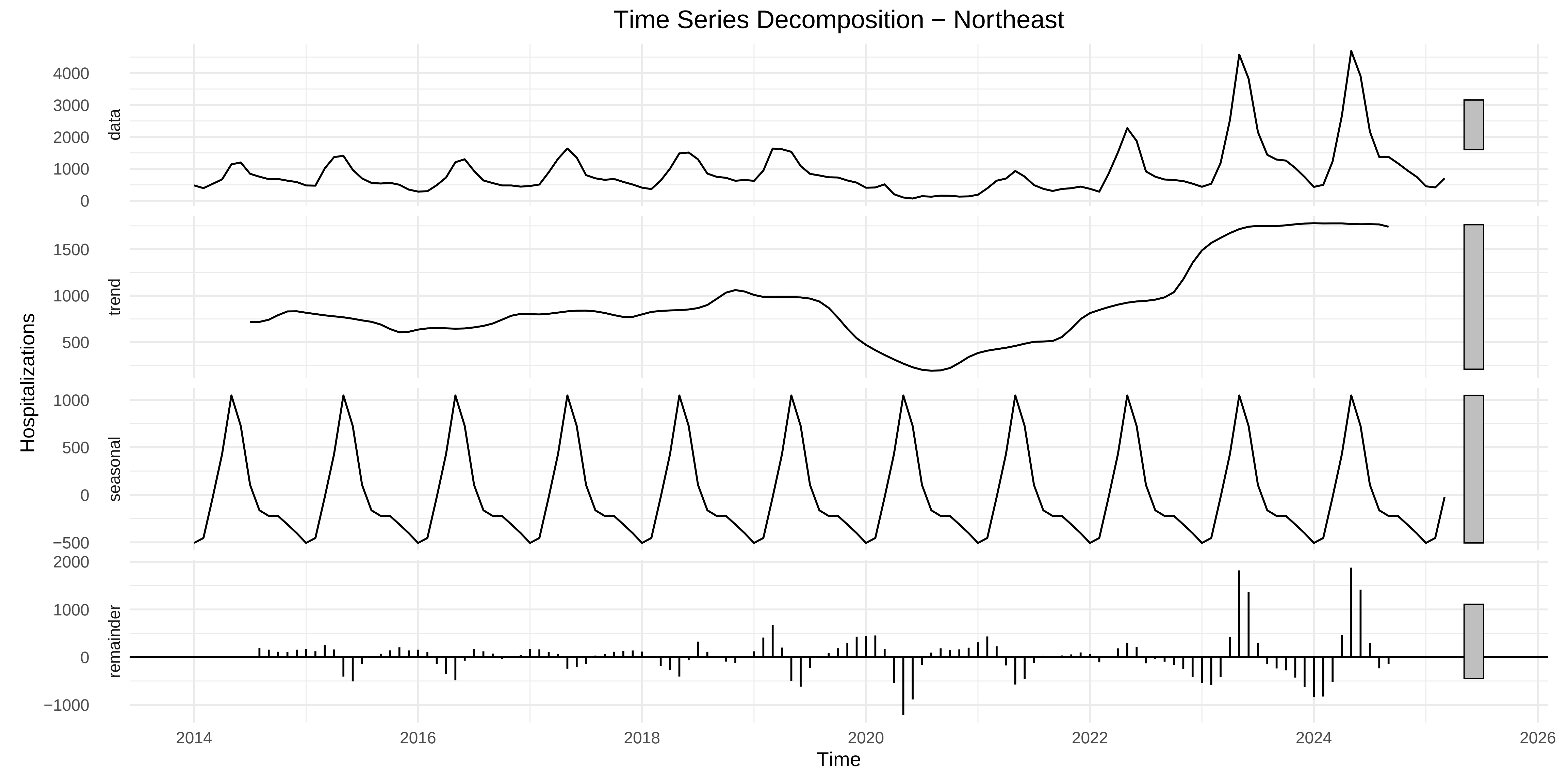
Time Series Decomposition − Northeast
Hospitalizations
data
trend
seasonal
remainder
4000
3000
2000
1000
0
1500
1000
500
1000
500
0
−500
2000
1000
0
−1000
2014
2016
2018
2020
2022
2024
2026
Time

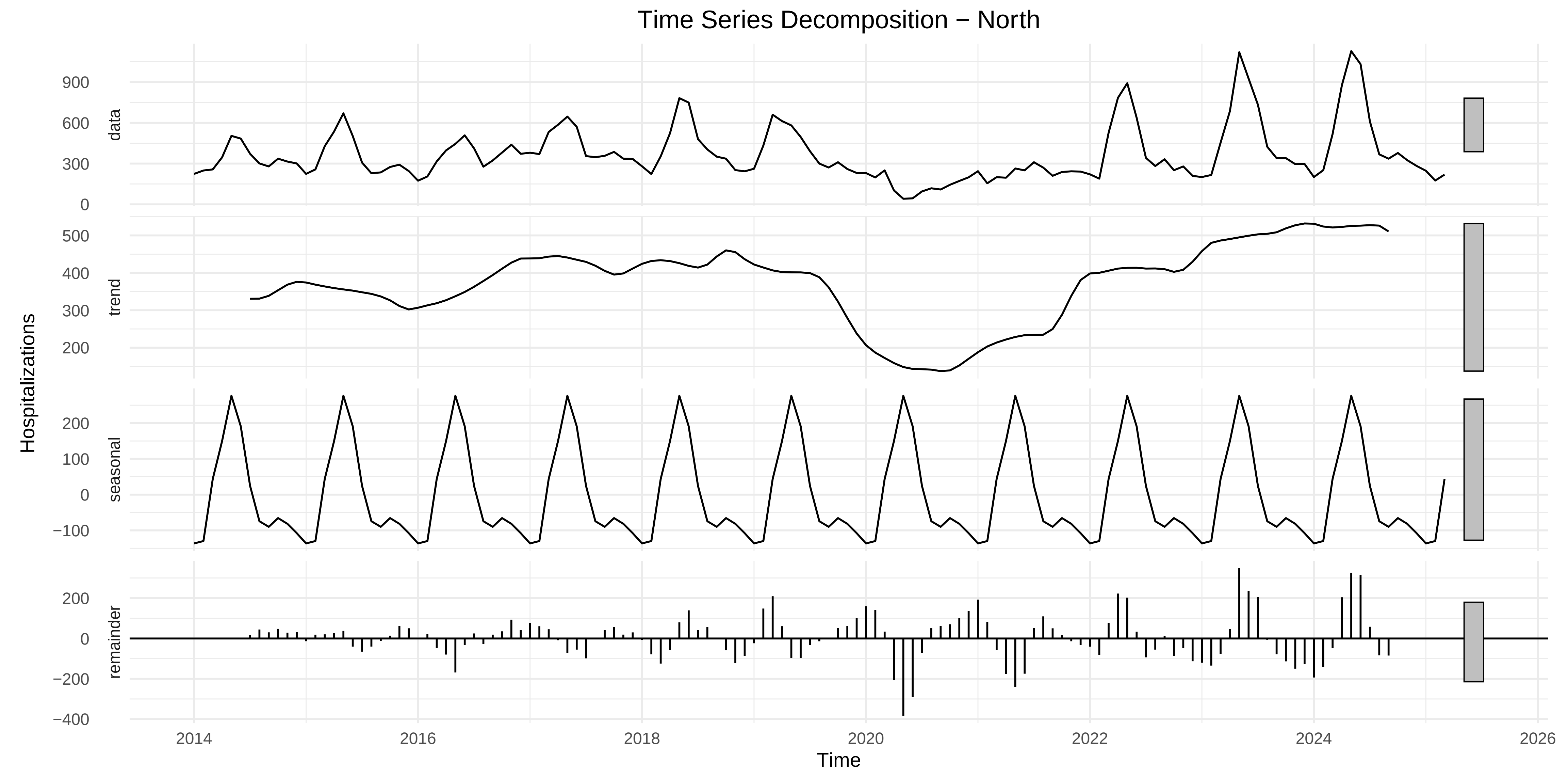
Time Series Decomposition − North
Hospitalizations
data
trend
seasonal
remainder
900
600
300
0
500
400
300
200
100
200
100
0
−100
200
0
−200
−400
2014
2016
2018
2020
2022
2024
2026
Time

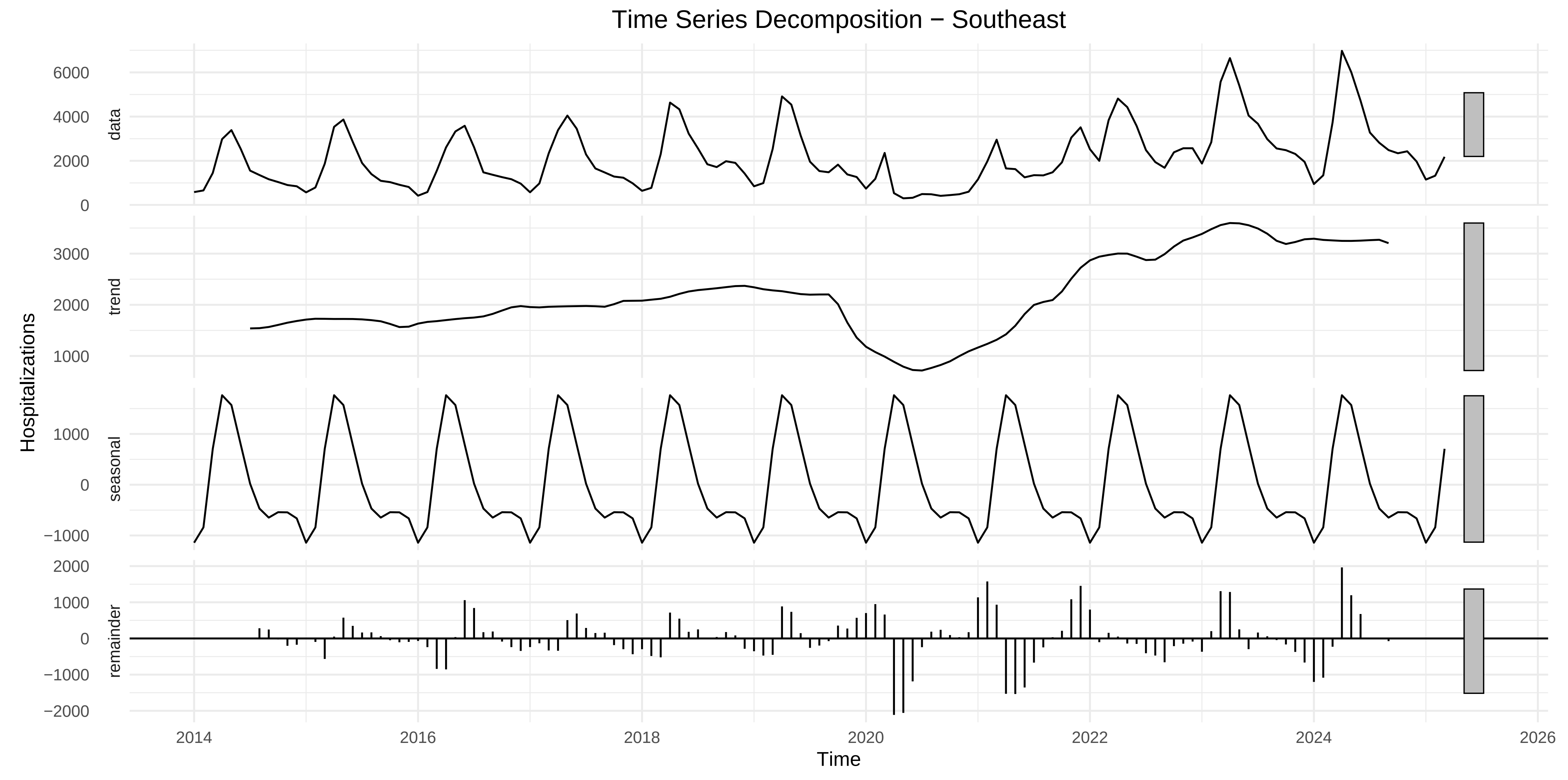
Time Series Decomposition − Southeast
Hospitalizations
data
trend
seasonal
remainder
6000
4000
2000
0
3000
2000
1000
1000
0
−1000
2000
1000
0
−1000
−2000
2014
2016
2018
2020
2022
2024
2026
Time

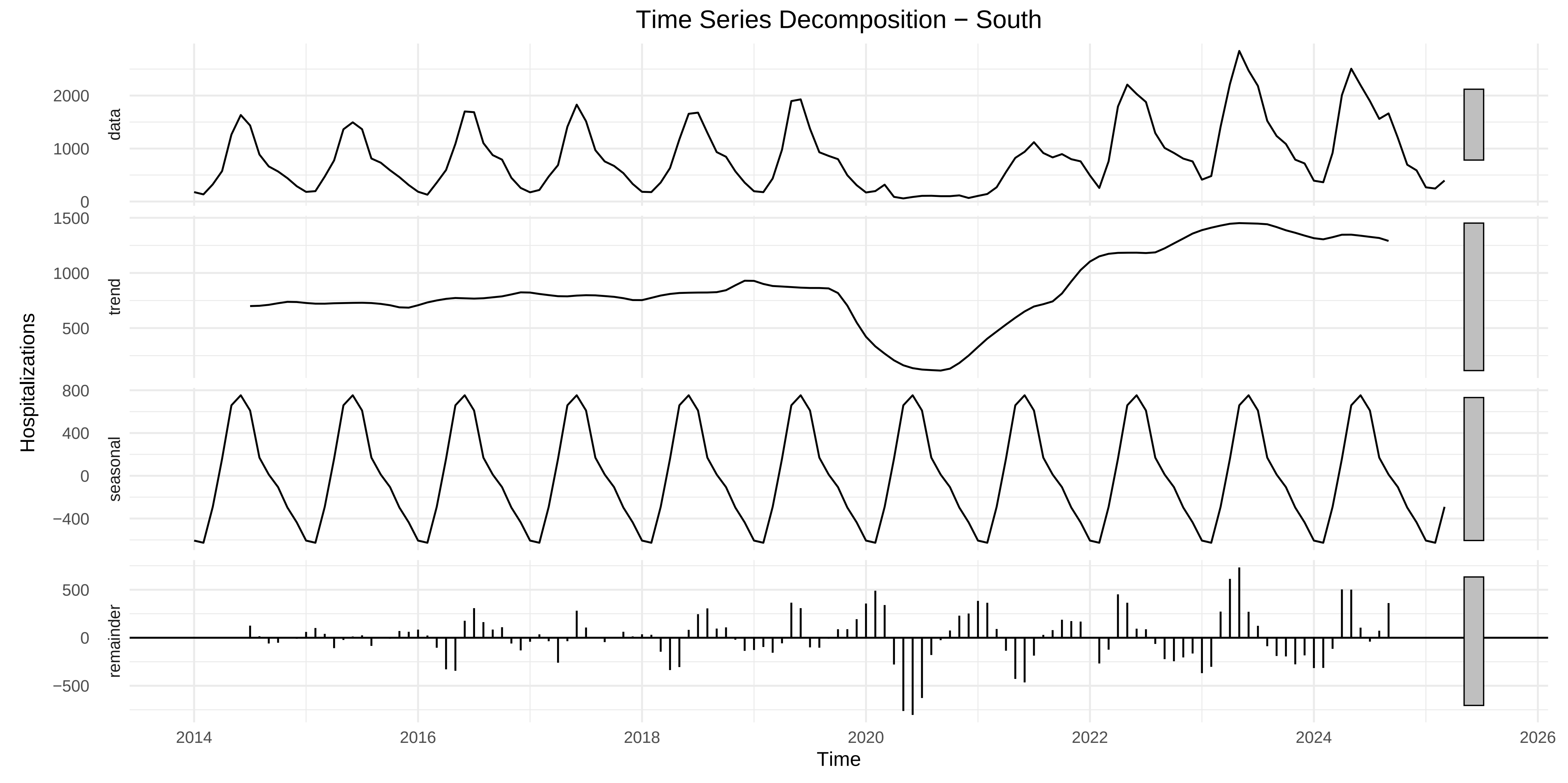
Time Series Decomposition − South
Hospitalizations
data
trend
seasonal
remainder
2000
1000
0
1500
1000
500
800
400
0
−400
500
0
−500
2014
2016
2018
2020
2022
2024
2026
Time
