# Supplementary material for: Trends and estimated costs of hospitalizations potentially associated with respiratory syncytial virus among children in Brazil, 2014-2025
Source: Rev Soc Bras Med Trop. 2026 Sep 18;59:e0120-2026. doi: 10.1590/0037-8682-0120-2026 (PMC13592856; doi:10.1590/0037-8682-0120-2026)
Supplement: Supplementary file 2 [file 1678-9849-rsbmt-59-e0120-2026-md2.pdf]

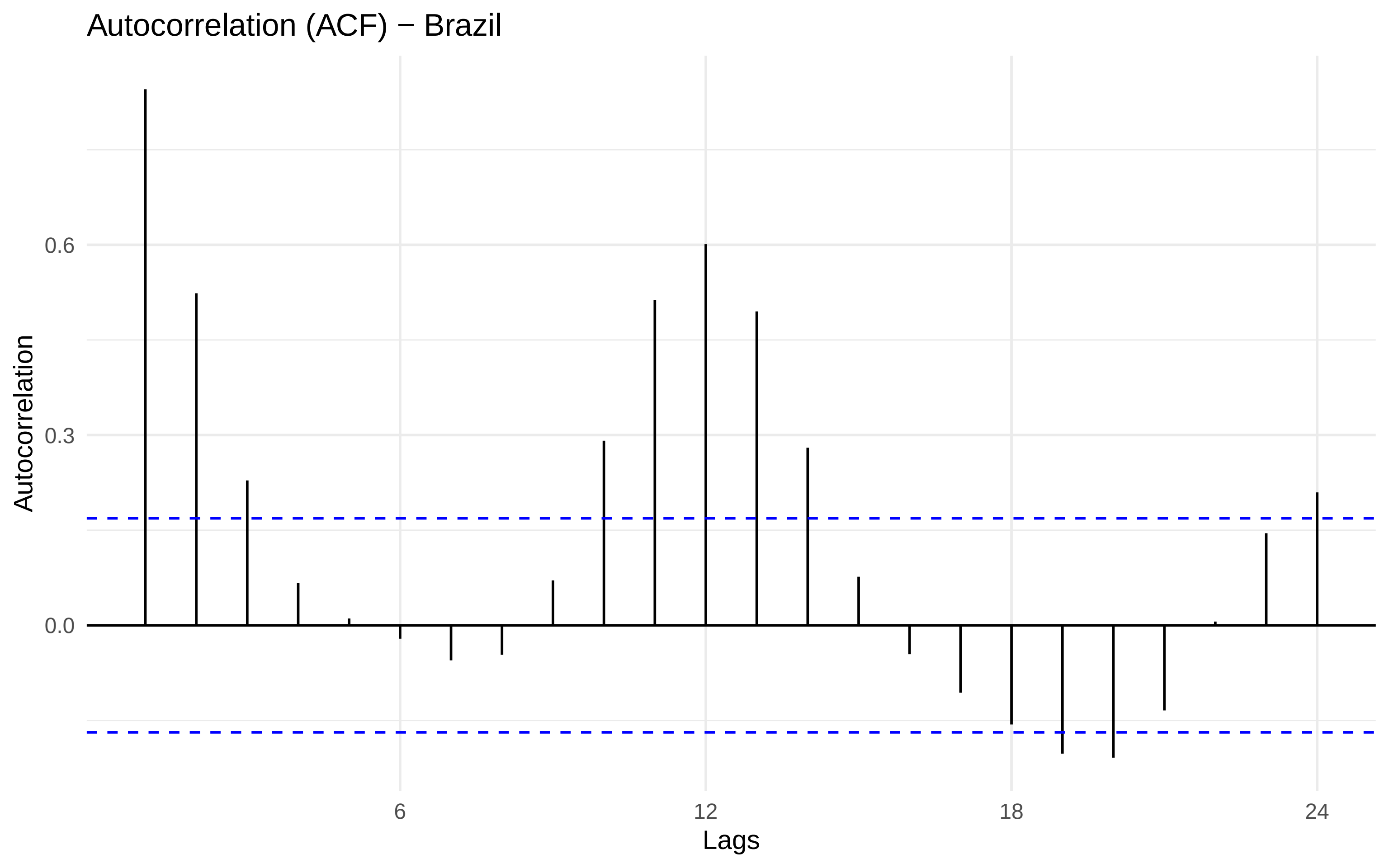
Autocorrelation (ACF) − Brazil
Autocorrelation
0.6
0.3
0.0
6
12
18
24
Lags

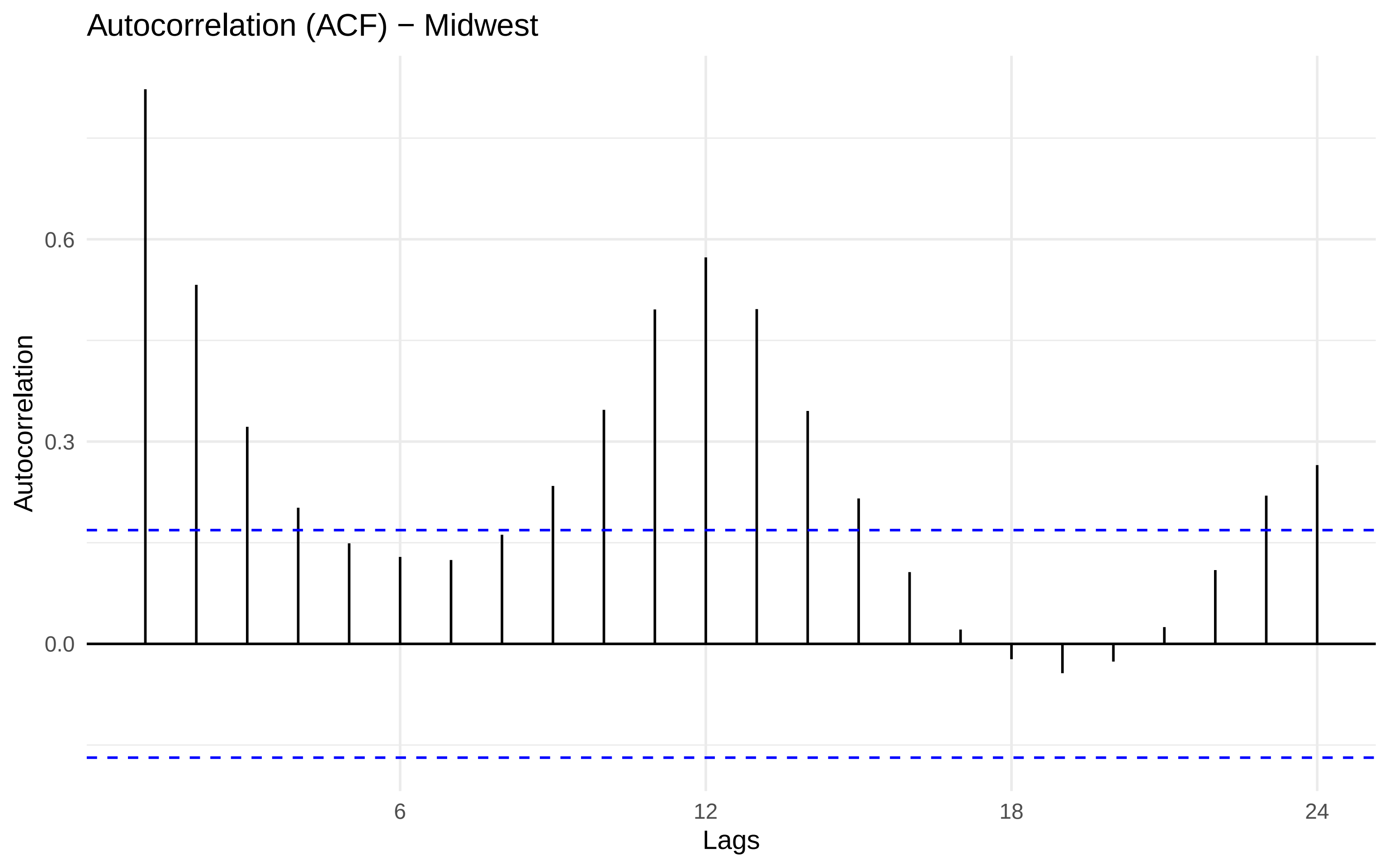
Autocorrelation (ACF) − Midwest
Autocorrelation
0.6
0.3
0.0
6
12
18
24
Lags

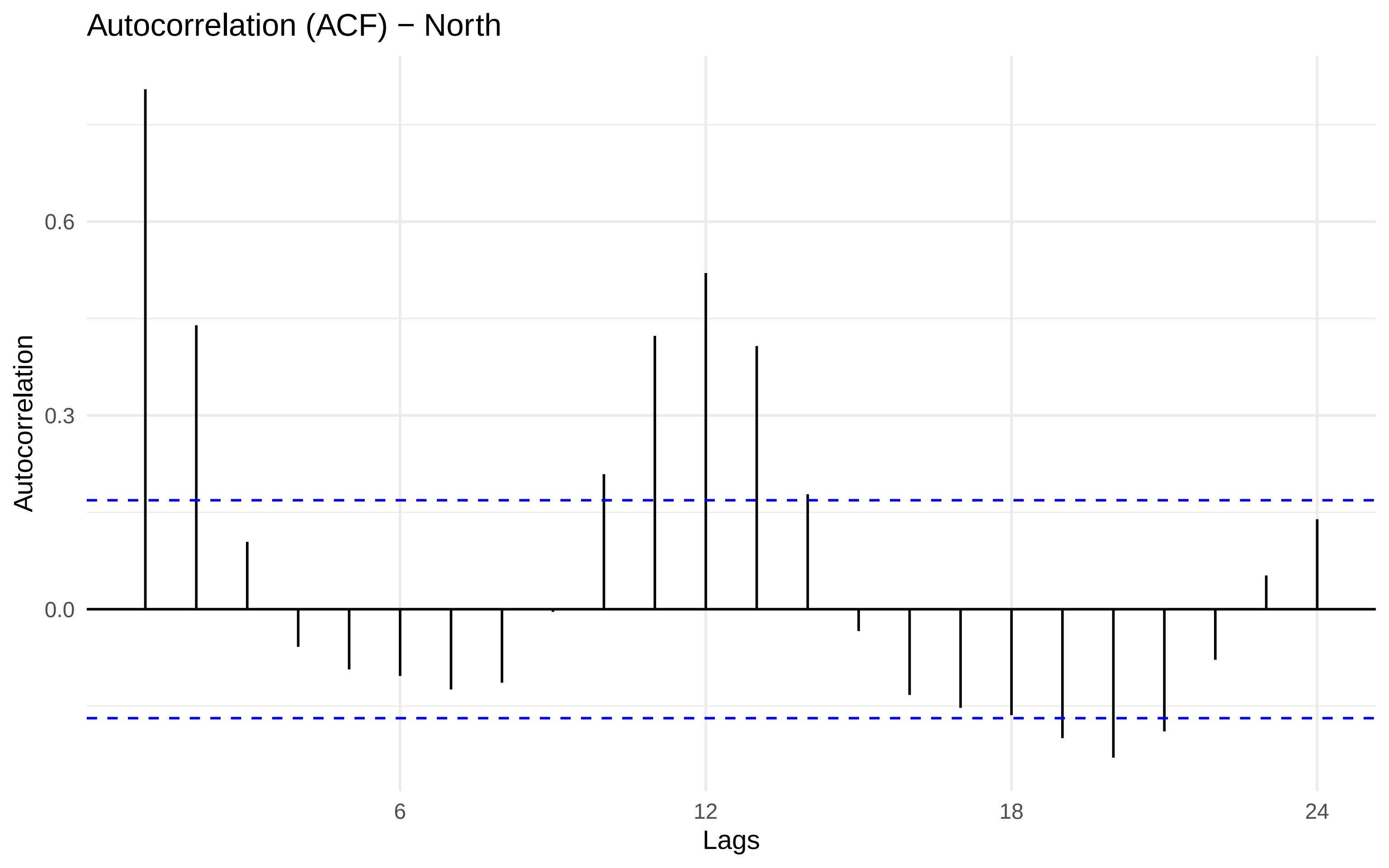
Autocorrelation (ACF) − North
Autocorrelation
0.6
0.3
0.0
6
12
18
24
Lags

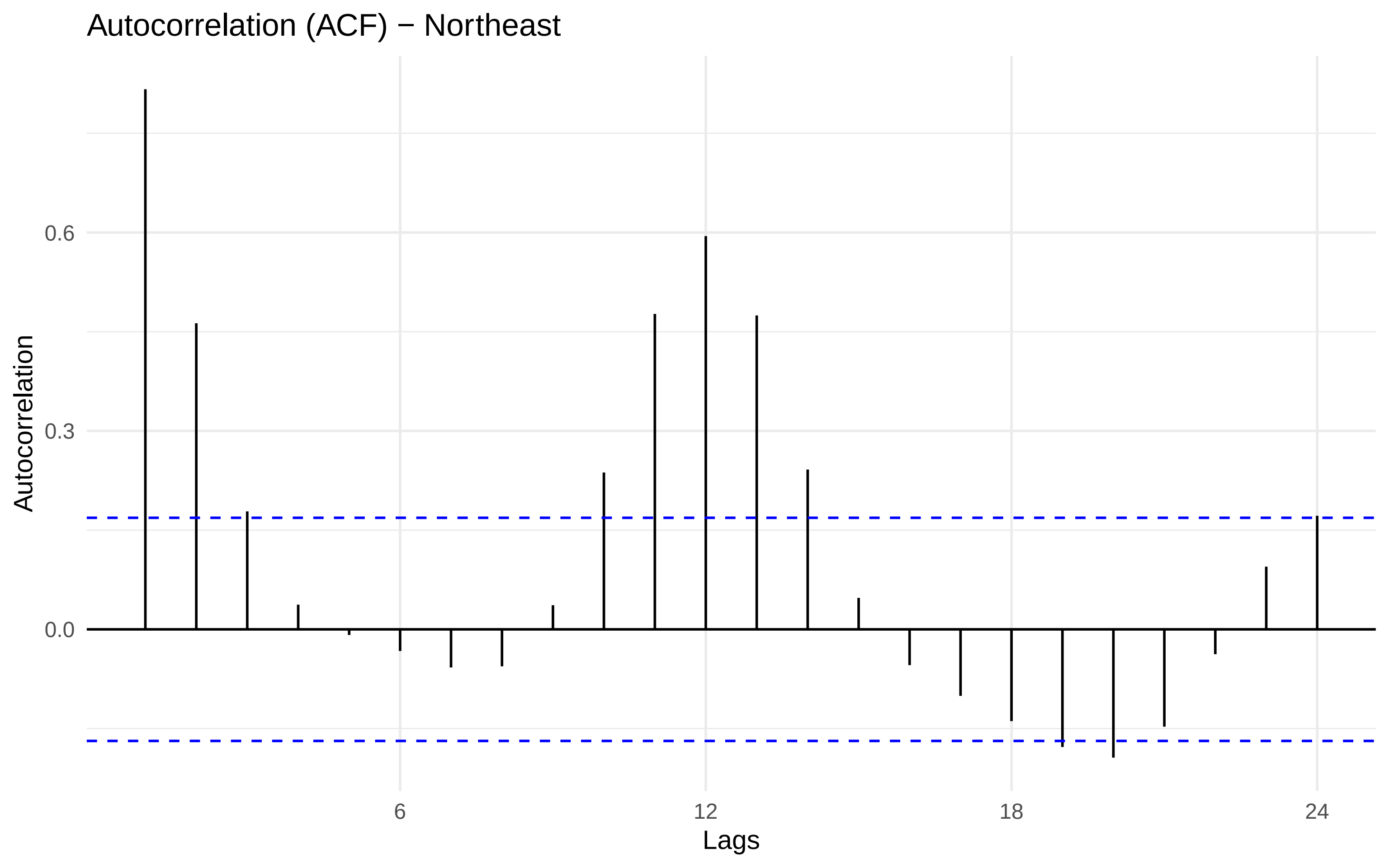

Autocorrelation (ACF) − Northeast
Autocorrelation
0.6
0.3
0.0
6
12
18
24
Lags

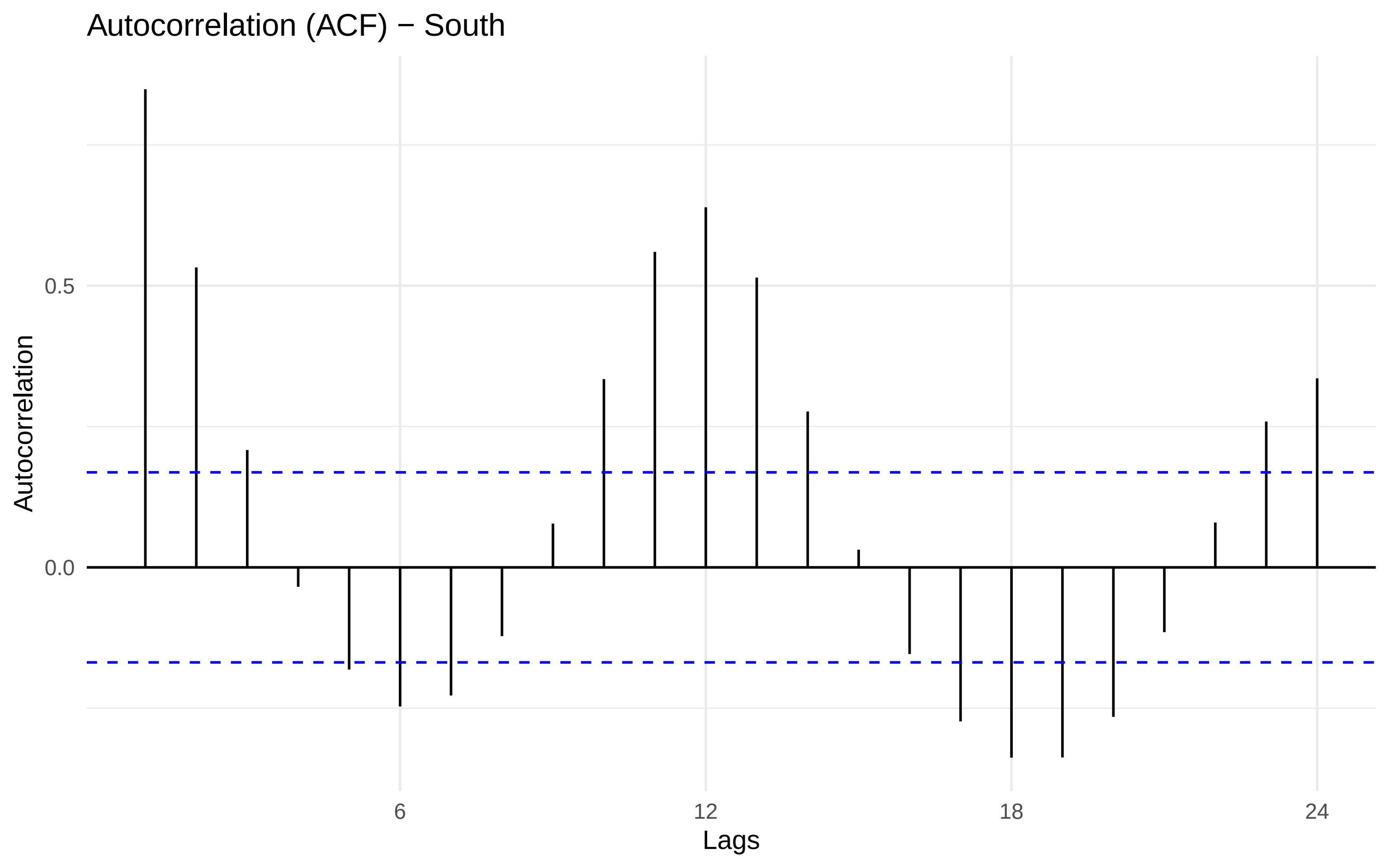
Autocorrelation (ACF) − South
Autocorrelation
0.5
0.0
6
12
18
24
Lags

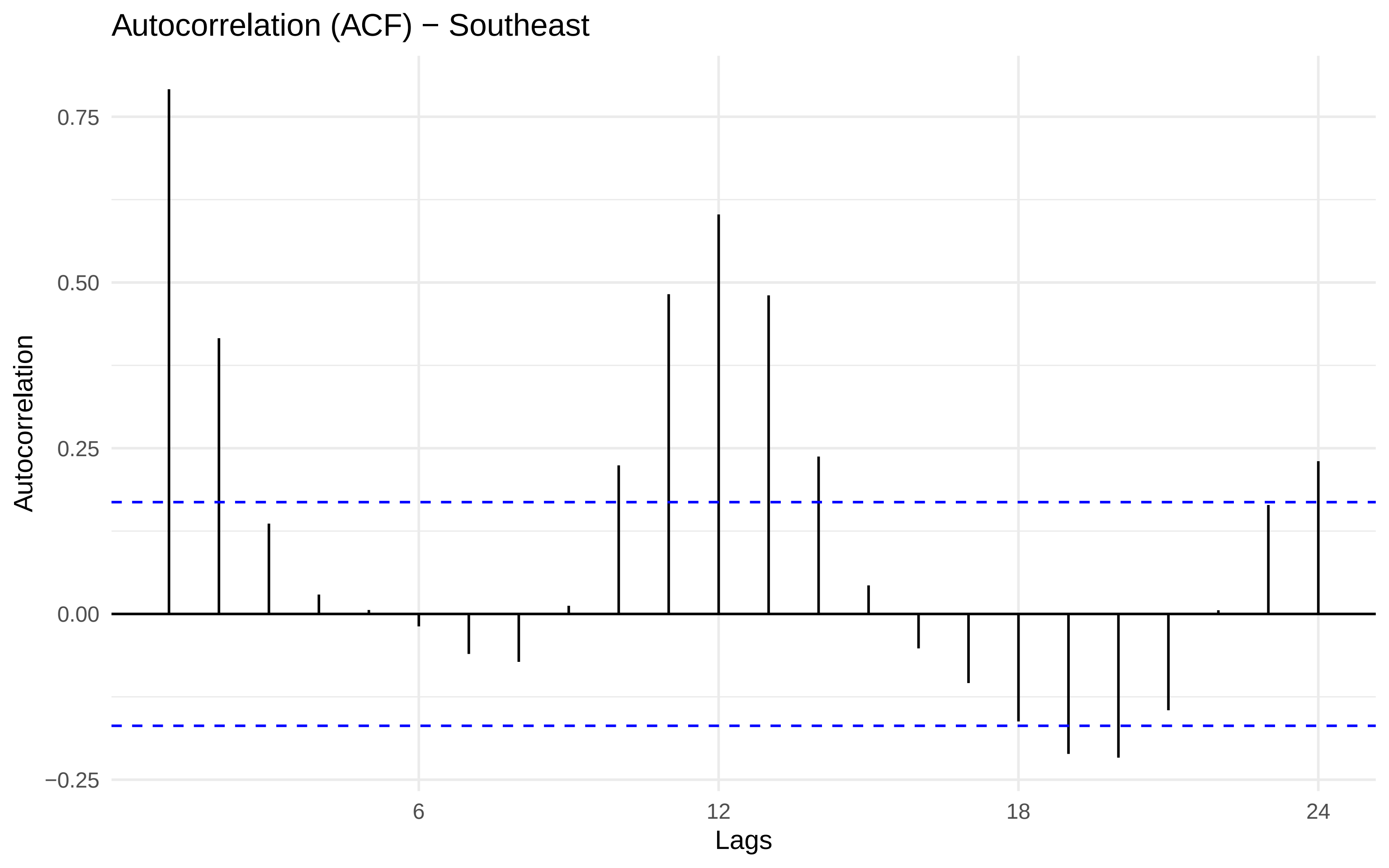
Autocorrelation (ACF) − Southeast
0.75
0.50
0.25
0.00
−0.25
Autocorrelation
6
12
18
24
Lags
