# Supplementary material for: Trends and estimated costs of hospitalizations potentially associated with respiratory syncytial virus among children in Brazil, 2014-2025
Source: Rev Soc Bras Med Trop. 2026 Sep 18;59:e0120-2026. doi: 10.1590/0037-8682-0120-2026 (PMC13592856; doi:10.1590/0037-8682-0120-2026)

Correlation: Length of Stay and Cost − Brazil

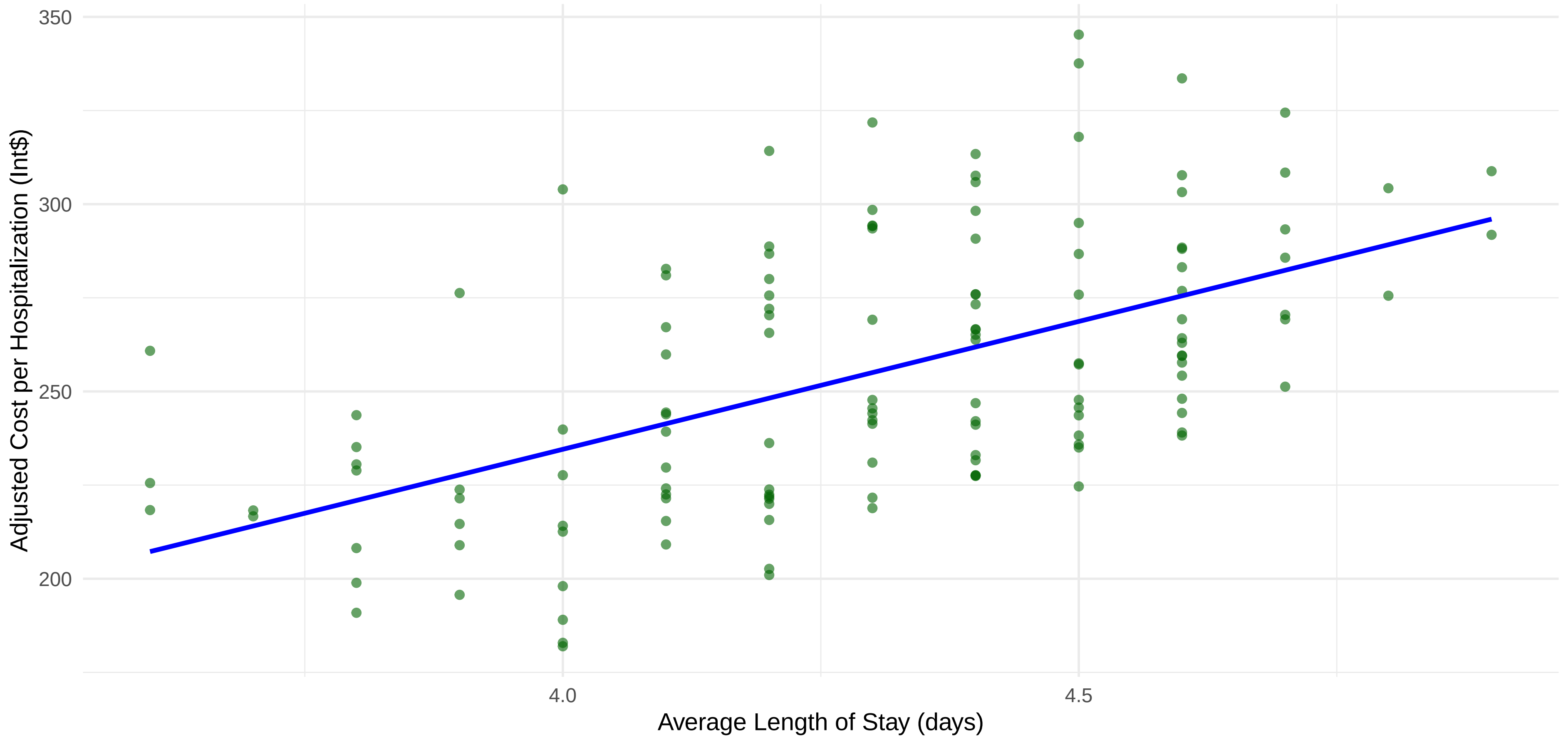


Correlation: Length of Stay and Cost − Midwest

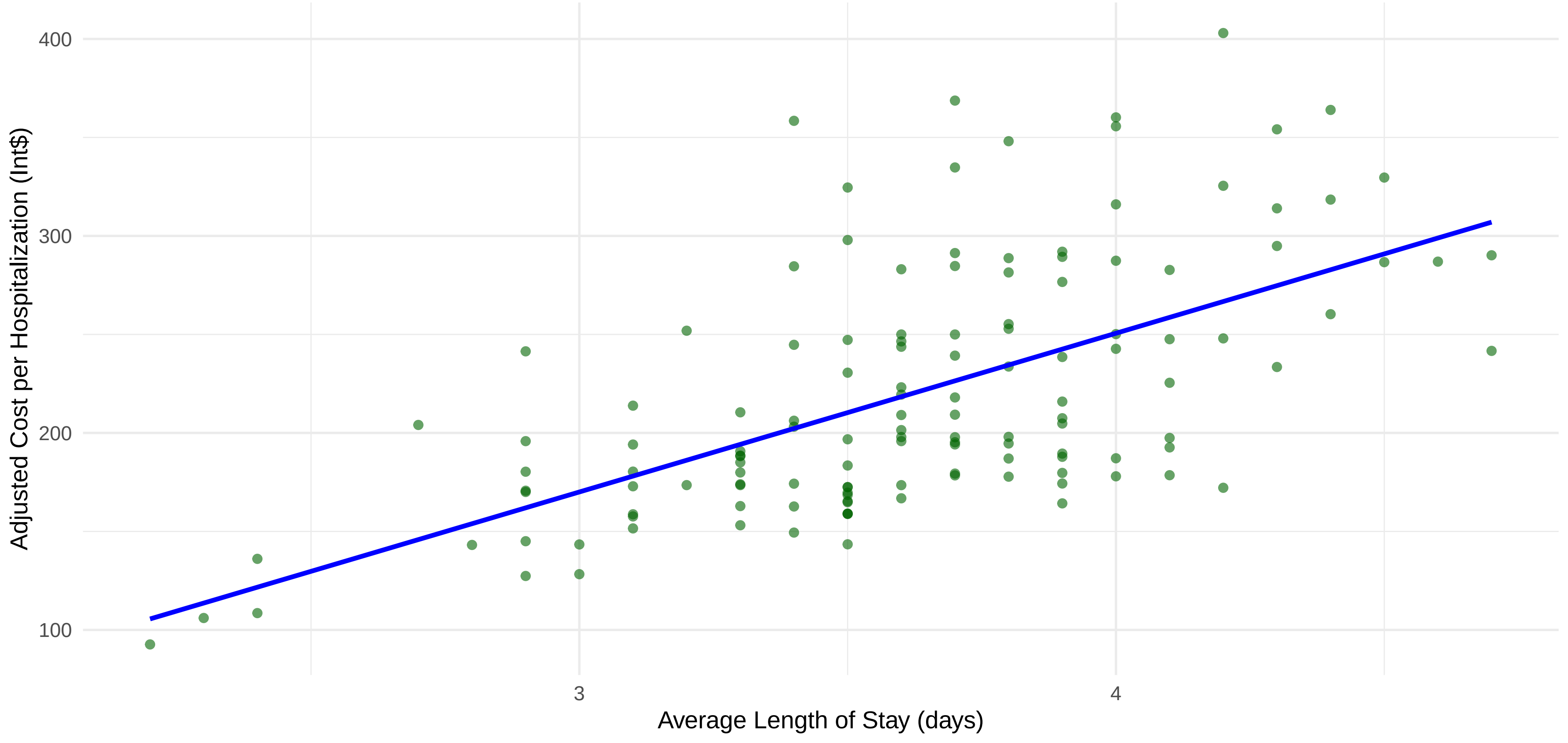


Correlation: Length of Stay and Cost − Northeast

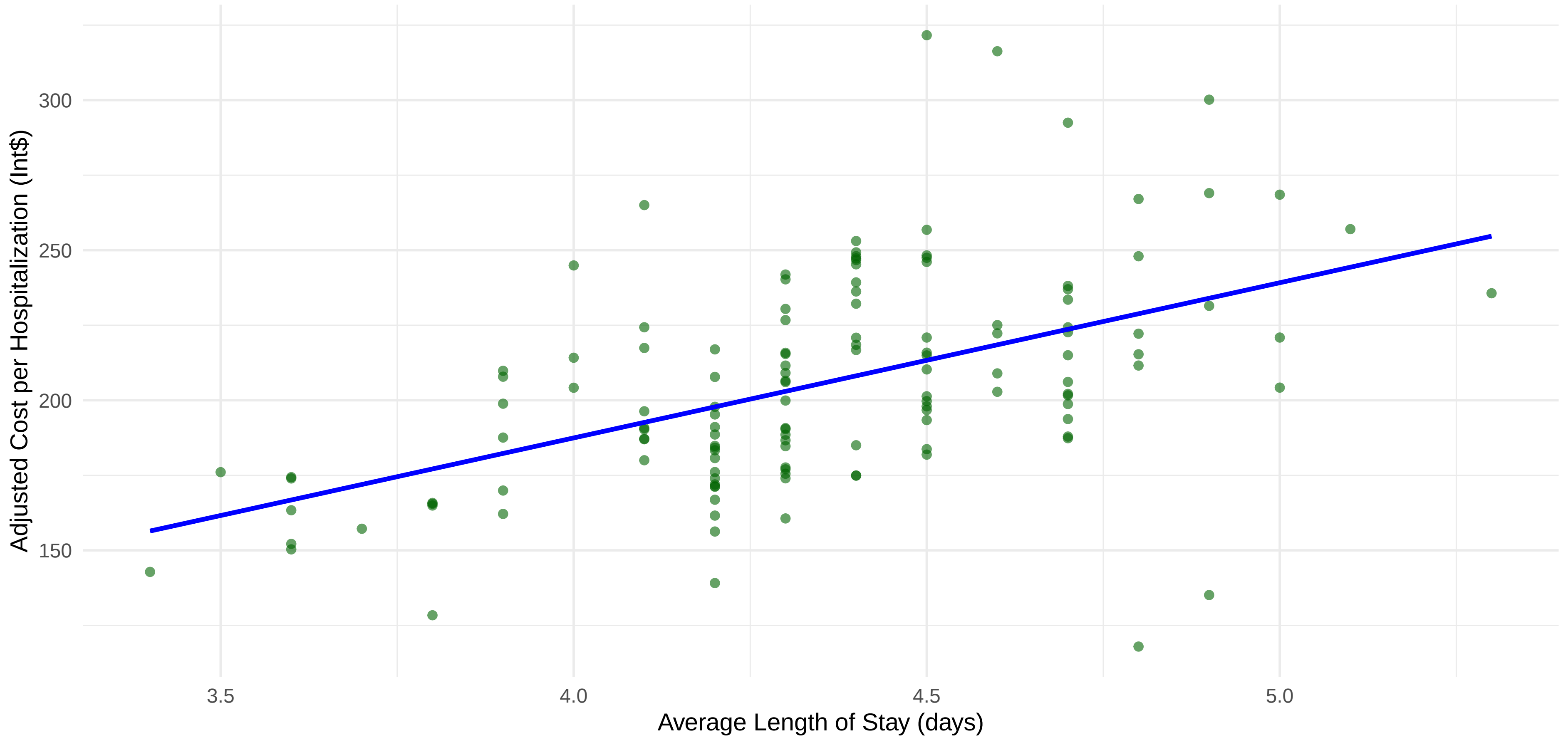


Correlation: Length of Stay and Cost − North

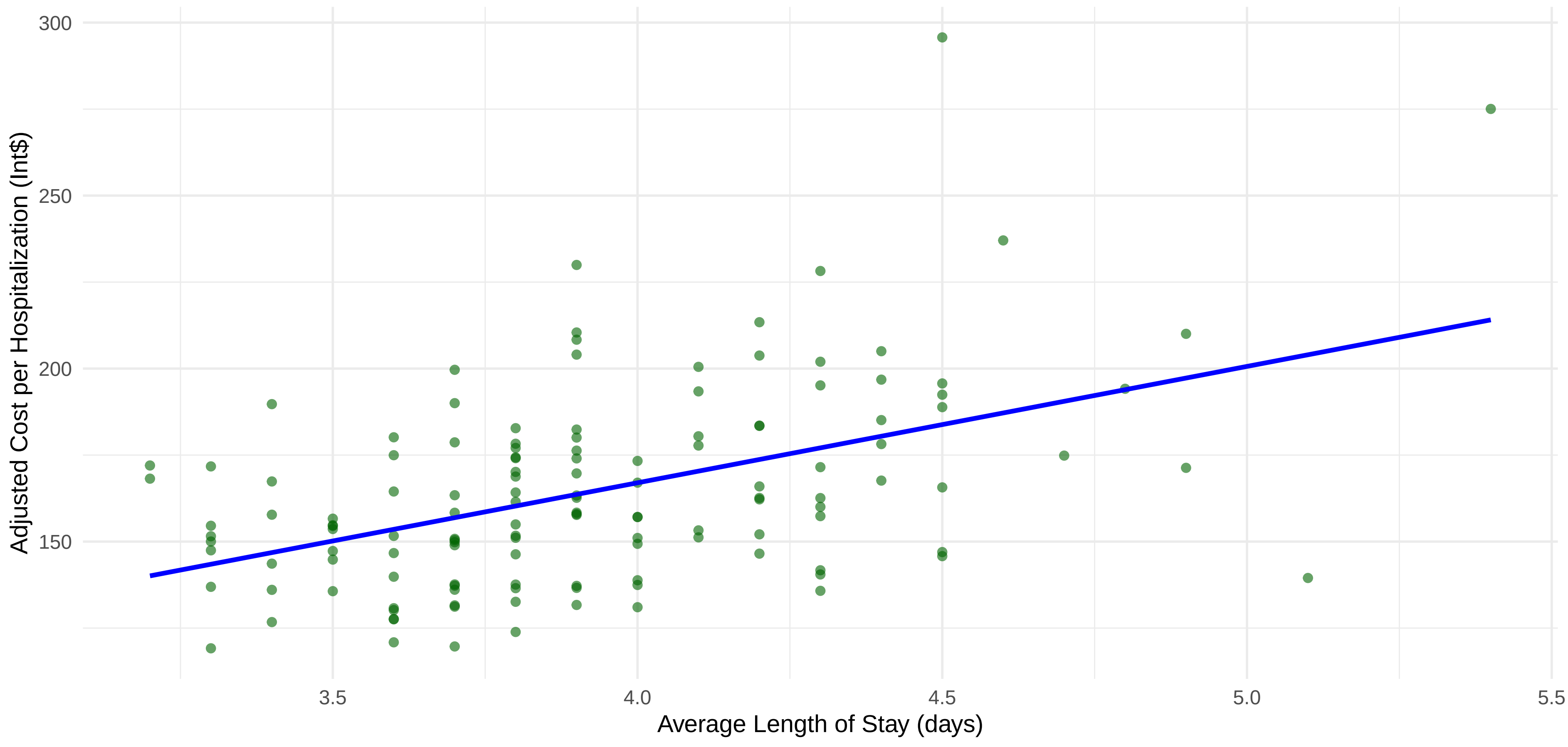


Correlation: Length of Stay and Cost − Southeast

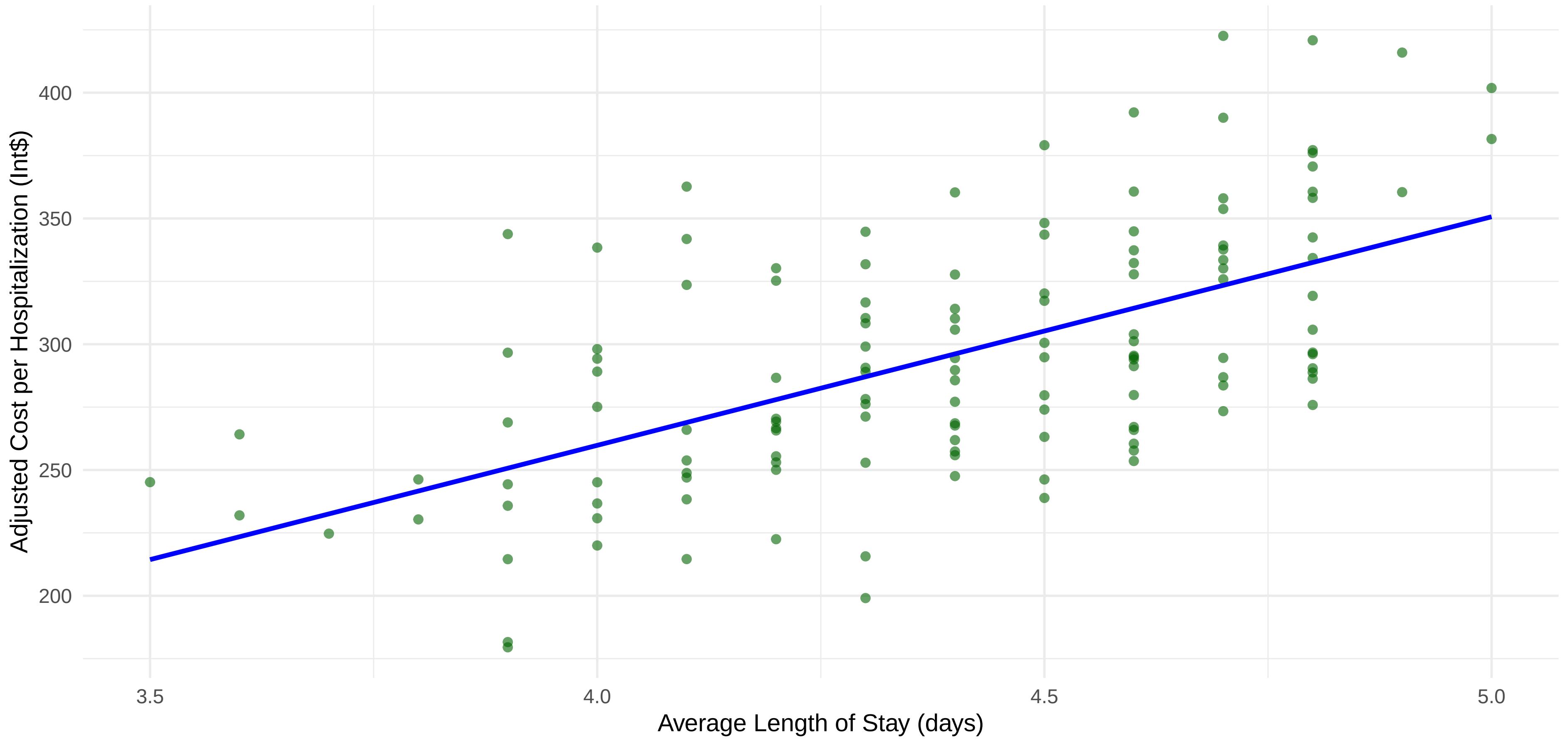


Correlation: Length of Stay and Cost − South

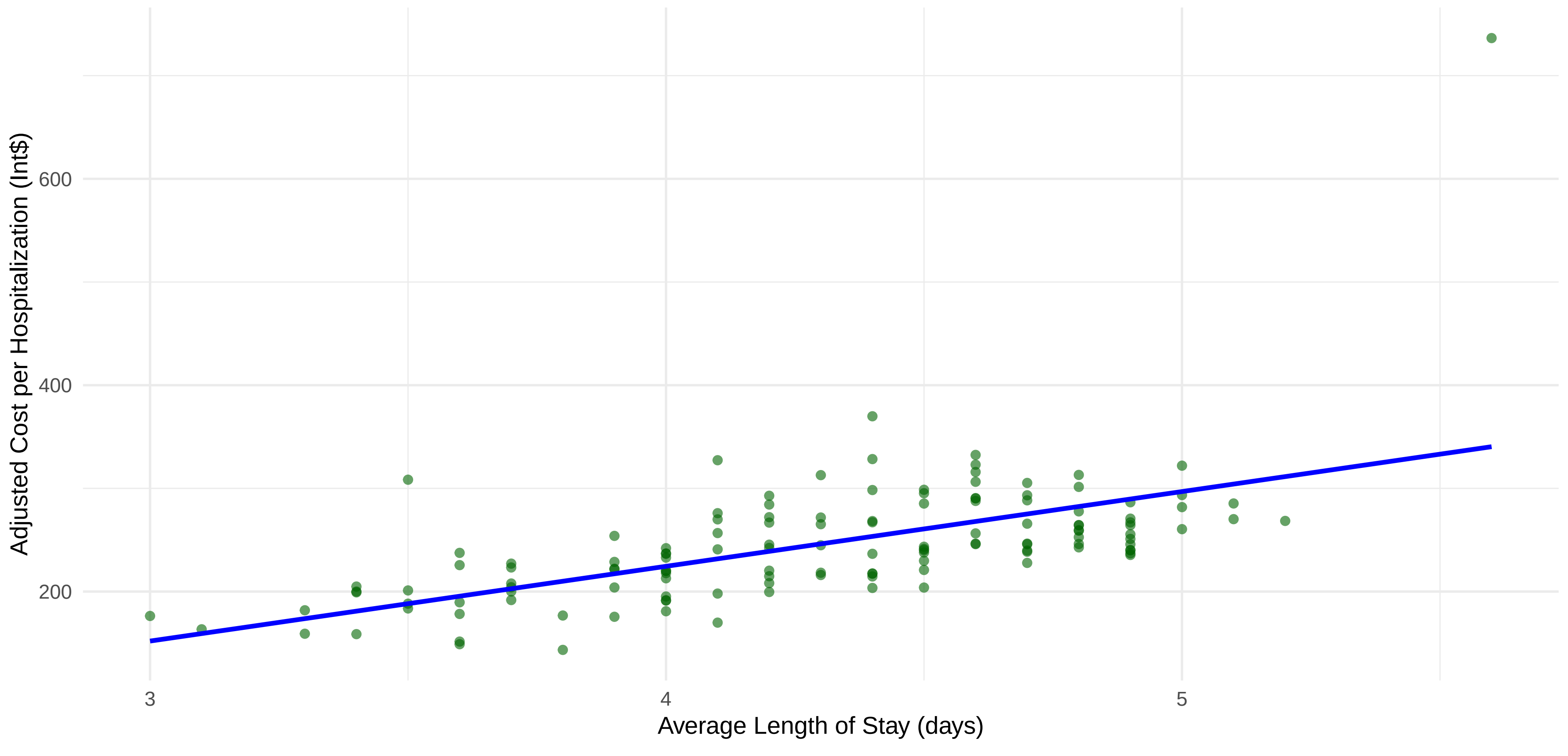

Supplement: Supplementary file 3 [file 1678-9849-rsbmt-59-e0120-2026-md3.pdf]
